# CD28/PD1 co-expression: dual impact on CD8^+^ T cells in peripheral blood and tumor tissue, and its significance in NSCLC patients' survival and ICB response

**DOI:** 10.1186/s13046-023-02846-3

**Published:** 2023-10-28

**Authors:** Belinda Palermo, Ornella Franzese, Giuseppe Frisullo, Lorenzo D’Ambrosio, Mariangela Panetta, Giulia Campo, Daniel D’Andrea, Isabella Sperduti, Francesca De Nicola, Frauke Goeman, Filippo Gallina, Paolo Visca, Francesco Facciolo, Paola Nisticò

**Affiliations:** 1 Tumor Immunology and Immunotherapy Unit, IRCCS-Regina Elena National Cancer Institute, Rome, Italy; 2 Department of Systems Medicine, University of Rome “Tor Vergata”, Rome, Italy; 3 Department of Biosciences, School of Science and Technology, Nottingham Trent University, Nottingham, UK; 4 Biostatistics and Scientific Direction, IRCCS-Regina Elena National Cancer Institute, Rome, Italy; 5 SAFU Unit, IRCCS-Regina Elena National Cancer Institute, Rome, Italy; 6 Thoracic-Surgery Unit, IRCCS-Regina Elena National Cancer Institute, Rome, Italy; 7 Pathology Unit, IRCCS-Regina Elena National Cancer Institute, Rome, Italy

**Keywords:** Non-small cell lung cancer, CD8+ T cells, PD-1, CD28, T-cell functionality, Single-cell RNA-Seq, Immune Checkpoint Blockade

## Abstract

**Background:**

Immune checkpoint blockade (ICB) has significantly prolonged survival of non-small cell lung cancer (NSCLC) patients, although most patients develop mechanisms of resistance. Recently single-cell RNA-sequencing (scRNA-Seq) revealed a huge T-cell phenotypic and (dys)functional state variability. Accordingly, T-cell exhaustion is recognized as a functional adaptation, with a dynamic progression from a long-lived “pre-exhausted stem-like progenitor” to a “terminally exhausted” state. In this scenario it is crucial to understand the complex interplay between co-stimulatory and inhibitory molecules in CD8^+^ T-cell functionality.

**Methods:**

To gain a baseline landscape of the composition, functional states, and transcriptomic signatures predictive of prognosis, we analyzed CD8^+^ T-cell subsets characterized by the presence/absence of PD1 and CD28 from periphery, adjacent non-tumor tissue and tumor site of a cohort of treatment-naïve NSCLC patients, by integrated multiparametric flow cytometry, targeted multi-omic scRNA-seq analyses, and computational pipelines.

**Results:**

Despite the increased PD1 levels, an improved PD1^+^CD28^+^ T-cell polyfunctionality was observed with the transition from periphery to tumor site, associated with lack of TIGIT, TIM-3 and LAG-3, but not with Ag-experienced-marker CD11a. Differently from CD28^+^ T cells, the increased PD1 levels in the tumor were associated with reduced functionality in PD1^+^CD28^−^ T cells. CD11a^high^, although expressed only in a small fraction of this subset, still sustained its functionality. Absence of TIGIT, TIM-3 and CTLA-4, alone or combined, was beneficial to CD28^−^ T cells. Notably, we observed distinct T_RM_ phenotypes in the different districts, with CD28^+^ T cells more capable of producing TGFβ in the periphery, potentially contributing to elevated CD103 levels. In contrast CD28^−^ T_RM_ mainly produced CXCL13 within the tumor.

ScRNA-seq revealed 5 different clusters for each of the two subsets, with distinctive transcriptional profiles in the three districts. By interrogating the TCGA dataset of patients with lung adenocarcinoma (LUAD) and metastatic NSCLC treated with atezolizumab, we found signatures of heterogeneous T_RM_ and "pre-exhausted" long-lived effector memory CD8^+^ T cells associated with improved response to ICB only in the presence of CD28.

**Conclusions:**

Our findings identify signatures able to stratify survival of LUAD patients and predict ICB response in advanced NSCLC. CD28 is advocated as a key determinant in the signatures identified, in both periphery and tumor site, thus likely providing feasible biomarkers of ICB response.

**Graphical Abstract:**

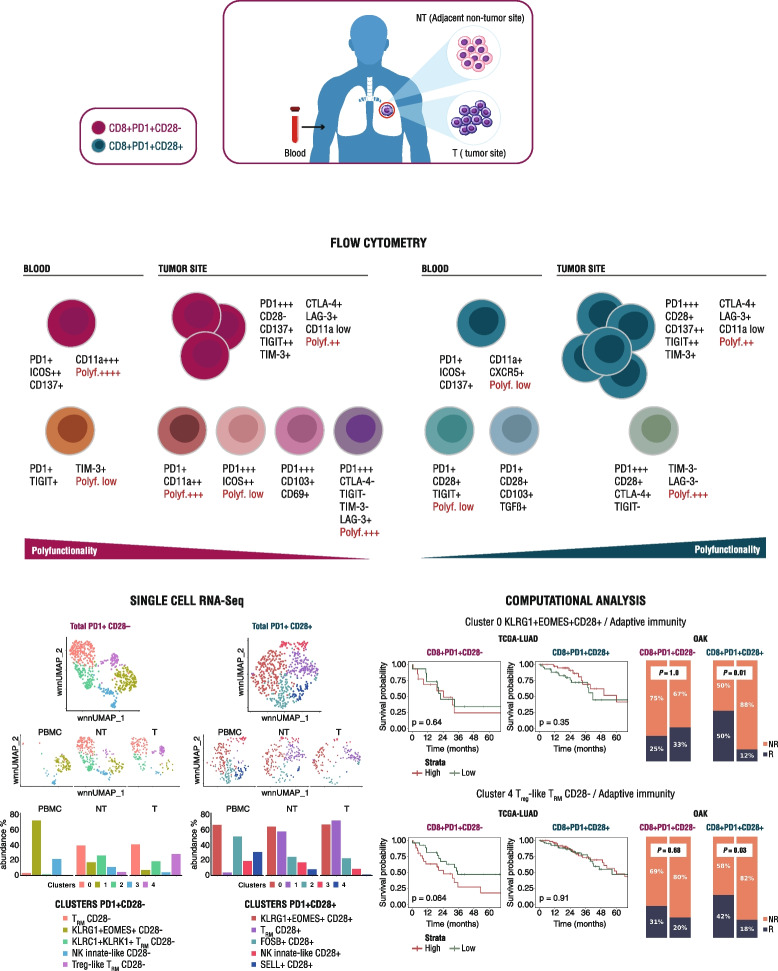

**Supplementary Information:**

The online version contains supplementary material available at 10.1186/s13046-023-02846-3.

## Background

Immune checkpoint blockade (ICB) has significantly prolonged survival of non-small cell lung cancer (NSCLC) patients [1–3], although only a minor percentage of the patients have a durable response. The challenge is to understand the complex mechanisms of resistance, recently highlighted thanks to the advent of single-cell RNA-based sequencing (scRNA-Seq) technology [4–6]. A huge variability in terms of phenotypic and (dys)functional states among the T-cell subsets, either circulating or infiltrating the tumor site has emerged [7, 8], fine-tuned by the complex network between T-cell differentiation state and local cues. Accordingly, exhaustion is currently recognized as an adaptation in terms of functional skills rather than a terminal condition of T-cell inactivation, with a more dynamic progression from a long-lived “pre-exhausted stem-like progenitor”, likely more responsive to immunological re-invigoration by ICB [9], to a “terminally exhausted” state [10]. Thus, it is imperative to define how T-cell differentiation states can vary between the periphery and the tumor site, where the microenvironment impacts both functionality and longevity of T cells.

A large subset of CD8^+^ T lymphocytes infiltrating the tumor site is represented by tissue-resident memory T cells (T_RM_) [11, 12], that are shaped by different cues from the local tissue, including Transforming Growth Factor (TGF) β [13, 14]. A high heterogeneity, defined by distinctive phenotypic and functional features in terms of chemokine receptors, adhesion, and effector molecules, has been observed among intra-tumor T_RM_ [15]. Accordingly, the clinical impact of this T-cell subset is still undefined, thus requiring a detailed analysis of its distinctive signatures [15, 16]. Notably, the unveiling of occurrence of T-cell subset with common signatures in both tumor site and peripheral blood is a major goal to allow longitudinal immune monitoring in clinical follow-up of cancer patients [5, 17].

Different patterns of expression and interplay between co-stimulatory and inhibitory molecules dictate T-cell functionality, and CD28 co-stimulatory molecule has been described as the primary target downstream of PD1 mediated inhibitory signaling [18, 19]. This is corroborated by the observation that CD28 is a prerequisite for an optimal T-cell re-invigoration following PD1 blockade [19–22].

In the present study, we in depth explored the CD8^+^ T-cell subsets characterized by the presence/absence of PD1 and CD28, by integrated multiparametric flow cytometry and multi-omic scRNA-Seq analyses in treatment-naïve NSCLC patients. To gain a baseline scenario of the composition, functional states, and transcriptomic signatures predictive of prognosis, we analyzed a cohort of NSCLC patients in periphery, adjacent non-tumor tissue (NT) and tumor site. We found distinctive and CD28-dependent functional states, differently distributed between the three districts analyzed, influenced by the interplay with specific co-stimulatory and inhibitory receptors (IRs) and defined by specific signatures. Searching for the predictive role of the identified profiles, we further interrogated TCGA datasets of lung adenocarcinoma (LUAD) and metastatic NSCLC patients treated with atezolizumab. Signatures of heterogeneous tumor infiltrating T_RM_ and “pre-exhausted” long-lived effector-memory CD8^+^ T cells were associated with better ICB response only in the presence of CD28.

Our findings identify gene expression profiles able to stratify survival of lung adenocarcinoma (ADC) patients and predict ICB response in advanced NSCLC. CD28 is advocated as a key determinant in CD8^+^ T cells with different functional states in the signatures identified, detected in both peripheral blood and tumor site, thus likely providing feasible biomarkers of ICB response.

## Methods

### Peripheral blood collection and tissue specimens

20 ml of blood were collected, after written informed consent, from 25 HDs, 52 NSCLC, 10 melanoma, and 10 PDAC patients, in EDTA vacutainer tubes (BD Bioscience, 367,864). Peripheral blood mononuclear cells (PBMC) were isolated using Ficoll-paque separation (Euroclone, DVCL5020) and cryopreserved in 10% Dimethyl sulfoxide (DMSO) (Sigma-Aldrich, D2650) until use.

Forty-three NSCLC patients were resected with curative intent at the Regina Elena National Cancer Institute during years 2013–2022. Main characteristics of the 68 NSCLC patients are listed in Table S1. Patients donated, after written informed consent, a portion of surgically resected tissue from distant non-tumoral (NT, derived from tissue isolated at least 5 cm away from the tumor core) and tumor site (T), for the tissue-infiltrating lymphocyte enrichment. Freshly resected tissues were processed within 20 min of removal from patient. Tissue samples were mechanically dissociated as small as possible under sterile conditions and placed into a humidified incubator at 37°C and 5% CO_2_ for 24-48h. Lymphocyte-enriched supernatant was collected, passed through a 70 µM nylon cell strainer (Miltenyi Biotec, 130–095-823) and immediately cryopreserved in 10% DMSO. For 32/68 total NSCLC patients, we were able to obtain samples matched for at least two districts among PBMC, NT and T site (Table S1).

### Melanoma cell lines

Melanoma cell lines Mel2 (HLA-A2^+^/Melan-A^−^gp100^−^) and Mel3 (HLA-A2^+^/Melan-A^+^gp100^+^) were kindly provided by Dr. A. Anichini (Fondazione IRCCS Istituto Nazionale dei Tumori, Milan, Italy), isolated from surgical specimens of tumors from patients as previously reported [23]. The cell lines were authenticated by chromosomal analysis (BMR Genomics, Italy) and routinely checked for mycoplasma using Mycoplasma PCR Reagent set (Euroclone, EMK090020). Cells were maintained in RPMI 1640 Medium (Euroclone, ECB9006L), supplemented with 10% fetal bovine serum (Euroclone, ECS5000L), 1% Glutamine (Euroclone, ECB3000D), 1% Penicillin/Streptomycin (Euroclone, ECB3001D), and cultured at 37°C in a 5% CO_2_ air-humidified atmosphere.

### iTAg™ MHC Tetramer staining and functional assessment of Ag-specific T-cell clones

Melan-A-specific and gp100-specific T-cell clones were generated by limiting dilution from pre-sensitized antigen (Ag)-specific T-cell lines, as described [23], and periodically re-stimulated in RPMI 1640 supplemented with 10% human serum (HS, noncommercial, prepared from healthy donors), 1 μg/ml PHA (Roche, 11,082,132,001), 25 U/ml recombinant IL-2 (rIL-2) (Miltenyi Biotec, 130–097-746), and 1 × 10^6^/ml irradiated allogenic PBMC as feeder cells. T-cell clones were used 13–15 d after stimulation. Ag-specificity of T-cell clones was assessed and periodically confirmed using anti-CD8-FITC and HLA-A*0201/PE-Melan-A (ELAGIGILTV) and HLA-A*0201/PE-gp100 (ITDQVPFSV) tetramer staining (see Table S2), performed for 30 min at room temperature (RT). Dead cells were excluded using propidium iodide staining (MP Biomedicals, 195,458). For intracellular staining of lytic molecules, Melan-A-specific and gp100-specific (*n* = 24) T-cell clones, isolated from 5 melanoma patients, were co-cultured with related (Melan-A^+^gp100^+^, Mel3) or unrelated (Melan-A^−^gp100^−^, Mel2) HLA/Ag melanoma cell lines, for 4–5 h at 37°C in the presence of protein transport inhibitor GolgiStop (BD, 554,724). T-cell clones were collected, and intracellular staining was performed using Intrasure kit (BD, 641,778) according to the manufacturer's instruction. Cells were then incubated for 30 min at RT with anti- PerCP-Cy5.5- tumor necrosis factor (TNF)-α, PE-Cy7-interferon (IFN)-γ and Alexa Fluor647-granzyme (GrzB) mAbs (see Table S2). Lytic activity was assessed in a standard 4-h ^51^Cr release assay. Mel2 and Mel3 melanoma cell lines were used as target cells and cytotoxicity was performed by incubating ^51^Cr-labeled target cells with effector cells (*n* = 26) at an effector: ratio (E:T) ratio of 20:1.

### RNA-Seq of Ag-specific T-cell clones

Total RNA was extracted from T cells using Qiazol (Qiagen, IT), purified from DNA contamination through a DNase I (Qiagen, IT) digestion step and further enriched by Qiagen RNeasy columns (Qiagen, IT). Integrity of RNA was assessed by Agilent 2100 Bioanalyzer (Agilent Technologies, CA). RNA libraries for sequencing were generated in triplicate using 500 ng of RNA for each sample according to the Illumina TruSeq Stranded Total RNA kit with an initial ribosomal depletion step by using Ribo Zero Gold (Illumina, CA). Libraries were sequenced in a paired-end mode (2 × 75 bp) with NextSeq 500 (Illumina, CA). For each sample generated by the Illumina platform, a pre-process step for quality control was performed to assess sequence data quality and to discard low-quality reads.

### Ag-specific T-cell clone RNA-Seq data analysis

RNA-Seq raw data were pre-processed using the nf-core/rnaseq pipeline (v2.4) [24] (quality check, demultiplexing, read mapping against GRCh37 and transcripts abundance quantification). Only genes with at least 10 total reads were selected for the downstream analysis. A Wald’s test was applied on the raw count’s matrix through the DESeq2 Bioconductor package (v1.34) [25] to identify differentially expressed genes (DEG) between groups (CD28^−^ and CD28^+^) (Table S3).

### In vitro T-cell activation and flow cytometry

PBMC, and cells from NT and tumor were thawed the day before staining and cultured overnight in RPMI 1640 supplemented with 10% HS and 12 U/ml rIL-2. The day after, 2–3 × 10^3^ unstimulated cells were stained with diverse combinations of mAbs specific for surface receptors (Table S2), for 30 min at 4°C. For some experiments, cells were washed and the intracellular staining with specific mAbs (i.e., versus CTLA-4, CXCL13, TGF-β) was performed using the Intrasure kit (BD Bioscience, 641,778), according to the manufacturer’s instruction. Ki67 expression was evaluated following 48 h of stimulation with plate-bound anti-CD3 mAb (2 μg/ml) (CBT3 IgG2a). For functional analyses, 2–3 × 10^5^ cells were activated with anti-CD3 mAb, in the presence of protein transport inhibitors Golgistop (BD 554724) and Golgiplug (BD 555029), for 5–6 h, before staining with diverse combinations of mAbs specific for surface and intracellular molecules (Table S2). For PD1 blocking experiments, cells were pretreated with functional grade purified anti-PD1 (IgG1, eBioscience 16–9989) (10 µg/ml) or control Isotype (IgG1, eBioscience 16–4714) mAbs, by overnight incubation. For some experiments, PBMC, NT and T cells were stimulated with plate-bound anti-CD3 mAb plus 25 U/ml rIL-2, for 48h or for 6 days, in the presence or absence of 10 ng/ml exogenous TGFβ1 (Bio-techne, 240-B-010/CF), according to an in-house dose–response setting, before staining with diverse combinations of mAbs specific for surface and intracellular molecules (Table S2). Intracellular staining was performed using Intrasure kit (BD Bioscience, 641,778) according to the manufacturer’s instruction, combining surface and intracellular mAbs (see Table S2). CMV-specific CD8^+^ T cells were identified using APC-conjugated HLA-A*0201 dextramer (Immudex, NLVPMVATV), for 30 min at RT. When feasible, dead cells were excluded using Fixable Viability Stain (BD, 565,388), for 10 min at room temperature before mAb staining. Cells were immediately acquired on BD FACSCantoII and BD FACSCelesta flow cytometers and analyzed by BD FACSDiva and Flowjo softwares.

### T-cell sorting and in-vitro expansion

PBMC from 5 HDs were thawed and cultured overnight in RPMI 1640 supplemented with 10% human serum (noncommercial, prepared from HDs). The day after, cells were stained and the CD8^+^PD1^+^CD28^−^ and CD8^+^PD1^+^CD28^+^ T-cell subsets were sorted by flow-cytometry (BD FACSMelody™ Cell Sorter). Approximately 20.000 to 200.000 sorted cells (for different samples) were stimulated in vitro with plate-bound anti-CD3 mAb plus 25 U/ml rIL-2, in the presence or absence of 10 ng/ml of exogenous TGFβ1 (according to the in-house dose–response setting) for 6 days.

### BD Rhapsody single-cell RNA-Seq

Single-cell RNA-Seq analysis was performed using BD Rhapsody™ Single-Cell Analysis System (BD Biosciences) on six samples, namely PBMC, NT- and tumor-infiltrating (T) cells from 2 NSCLC adenocarcinoma (ADC) patients. Cells were thawed and cultured overnight in RPMI 1640 supplemented with 10% human serum. The following day, cells were washed, and lymphocytes were sorted (BD FACSMelody™) according to their dimensions (FSC, forward-scatter and SSC, side-scatter parameters). Lymphocyte purity was evaluated on PBMC by the expression CD45 (Table S2) and was > 99%. The rationale for this approach was to avoid the potential interference of flow cytometry mAbs with Rhapsody mAbs, according to guidelines provided by the manufacturer.

Approximately 35.000 to 350.000 sorted cells (for the different six samples) were cultured overnight in RPMI 1640 supplemented with 10% human serum plus 25 U/ml rIL-2. On day 3 from thawing, according to the conditions employed in the multiparametric flow cytometry experiments, cells were activated with plate-bound anti-CD3 mAb (2 μg/ml) for 4h, then harvested and counted by trypan blue exclusion to perform single-cell analysis following the manufacturer’s protocol. Briefly, CD3-mAb activated cells were resuspended in 180 ul of Stain Buffer (BD, 554,656) and labeled with sample tags (BD, Hu Single Cell Sample Multiplexing Kit, 633,781) by incubation for 20 min at RT. Then, cells were washed four times with 2 ml of Stain Buffer and centrifuged for 5 min at 400xg. Next, cells were resuspended in 200 µl cold Sample Buffer (Rhapsody Cartridge reagent kit, 633,731) and counted by trypan blue exclusion. An equal number of labeled cells from each sample (20.000) were pooled together, washed with 1.5 ml of cold Sample Buffer, resuspended in 100 µl of Stain Buffer containing the Fc-block (BD, 564,220) and incubated for 10 min at RT. Then, pooled cells were combined with the AbSeq Ab-Oligos (BD Immune Discovery Panel, 625,970), plus three custom supplemental mAbs (Hu CD103 Ab-O, 940,067; Hu CD39 Ab-O,940,073; Hu CD11a Ab-O, 940,077), and incubated for 30 min on ice. Cells were washed three times, resuspended in 620 µl cold Sample Buffer and counted with dispensable hemocytometer in the Rhapsody apparatus. 40.000 cells were loaded onto BD Rhapsody cartridge (633,733) and incubated for 15 min at RT. Then, Cell Capture Beads (Rhapsody Cartridge reagent kit, 633,731) were loaded onto the cartridge (Rhapsody Cartridge Kit, 633,733) and incubated 3 min at RT. Cartridge was washed, cells were lysed, and Cell Capture Beads were recovered and washed prior to performing reverse transcription and treatment with Exonuclease I (Rhapsody cDNA Kit, 633,773). cDNA libraries were prepared on 16.000 cells using the Immune Response Panel Hs (BD, 633,750; Rhapsody Targeted mRNA & AbSeq Amp Kit; 633,774) consisting of 399 genes. Briefly, three different cDNA libraries were prepared following the protocol of the “mRNA Targeted, Sample Tag, and BD™ AbSeq Library Preparation” using the BD Rhapsody Targeted mRNA and AbSeq Amplification Kit. The first library includes the targeted transcriptome of the Immune Response Panel Hs for each cell. The second library encodes the sample tag which enables to assign each cell to the correct initial sample (Sample Tag). The third library contains the information of the proteins that were recognized on each cell by the Ab cocktail described above (AbSeq). The quality of the final indexed libraries was controlled using the Agilent High Sensitivity DNA Kit and the Bioanalyzer. The quantification was performed by Qubit fluorometer and the 1X dsDNA HS Assay Kit (Thermofisher Scientific) and by qPCR. The libraries were sequenced on the NextSeq 500 and NovaSeq 6000 instrument (Illumina) sequencing 64bp for Read 1, 48bp for Read 2 and 8bp for the index. The samples were sequenced to obtain a sequencing depth of 9000 read-pairs/cell for the targeted RNA panel, 800 read-pairs/cell for the Sample Tag and 19,000 read-pairs/cell for the 33 different AbSeq Ab-Oligos.

### BD Rhapsody single-cell data analysis

Raw multimodal scRNA-Seq data were pre-processed (quality check, demultiplexing, read mapping and generation of raw counts matrix) through the BD Rhapsody Targeted Analysis Pipeline on the SevenBridges web platform (https://www.sbgenomics.com). CD8^+^PD1^+^CD28^±^ T cells were manually gated on the SeqGeq application and selected for the following analyses. The raw counts matrix of the selected cells was imported in the R stastical environment, and all downstream analyses were performed using the “Seurat” (v4) R package. Raw UMI counts for transcript probes and AbSeq probe counts were normalized and scaled through the NormalizeData (“LogNormalize” method for UMI counts, “CLR” method for AbSeq as suggested by package developers) and ScaleData functions. Variable features and PCA were computed using the “FindVariableFeature” and “RunPCA” functions with default parameters. Subsequently, the integrative analysis of the two modalities was performed using weighted nearest neighbor (WNN) graphs following the standard CITE-Seq workflow from the “Seurat” package reference. UMAP embeddings and clustering were performed using the “runUMAP” and the “FindClusters” functions, respectively. UMAP visualizations were generated using the “DimPlot” function. For each cluster of interest (i.e., C0: KLRG1^+^EOMES^+^ CD28^+^, C2: KLRC1^+^KLRK1^+^T_RM_ CD28^−^, C4: Treg-likeT_RM_ CD28^−^) (Table S4 and Table S5), the marker genes were filtered to include the adaptive immunity-related genes with Fold Change > 1.5 and adjusted *P* value < 0.001. The list of 816 adaptive immune-related genes was derived from the Reactome database (“adaptive immunity” pathway, release 85) and downloaded using PathwayBrowser (version 3.7). As result, the signatures included 11 genes for the KLRG1^+^EOMES^+^CD28^+^ (Adaptive Immunity C0 signature: CD74, PTPRC, ITGA4, KLRG1, CD8A, TRAT1, CD8B, LCK, CD3E, HLA-DMA, HLA-DPA1), 7 genes for KLRC1^+^KLRK1^+^T_RM_ CD28^−^ (Adaptive Immunity C2 signature: KLRC1, CD8A, CD8B, CD3E, KLRK1), and 6 genes for Treg-likeT_RM_ CD28^−^ (Adaptive Immunity C4 signature: CD74, LAG3, CD8B, HLA-DMA, HLA-DRA, HLA-DPA1).

### Methods for patients' stratification

To investigate the impact of T-cell subpopulations of interest on Lung ADC patients’ prognosis and response to ICB, we interrogated TCGA-LUAD and OAK datasets, respectively. For both datasets, patients with Overall Survival (OS) > 1 month were considered for the analysis. For the OAK dataset, only Lung ADC samples that underwent ICB therapy (atezolizumab) were selected. To resemble tumor lymphocyte infiltration and ensure the presence of CD8^+^PD1^+^ T cells, patients were stratified based on CD8 and PD1 gene expression levels. Patients with both CD8 and PD1 genes expression above the 25th percentile were considered as CD8^+^PD1^+^ cohort and, subsequently, stratified in two groups, CD28^+^ and CD28^−^, based on CD28 gene expression levels, considering the 25th percentile as cutoff.

### Survival analysis

Survival analysis for the TCGA-LUAD dataset was performed on the three different cohorts: CD8^+^PD1^+^, CD8^+^PD1^+^CD28^−^ and CD8^+^PD1^+^CD28^+^, using the “survival” and “survminer” packages in the R statistical environment (v4.1.2). The average of the log2-scaled expression values from the signature genes were used as scores for the respective gene signatures. Patients were stratified into two groups based on the levels of signature expression, using the median value as threshold and removing a percentage of patients near to the median value (i.e*.,* 0%, 10% or 50%). Patients with a signature score above the upper threshold were stratified as “High” while patients with a signature score below the lower threshold were stratified as “Low”. Survival curves were estimated using the Kaplan–Meier method, and statistical differences were tested using the log-rank test. *P* values < 0.05 were considered statistically significant.

### Therapy response analysis OAK

To analyze the correlation of each signature with the response to immunotherapy on the OAK dataset, patients were stratified as for the TCGA-LUAD dataset. The average of the Z-scored normalized expression values from the signature genes were used as scores for the respective gene signatures. Patients were stratified into two groups based on the levels of signature expression, using the median value as threshold and removing a percentage of patients near to the median value (i.e. 0%, 10%, or 50%). Patients with a signature score above the upper threshold were stratified as “High” while patients with a signature score below the lower threshold were stratified as “Low”. Then, relative signature levels were plotted against Clinical Response, considering patients with Partial Response (PR) or Complete Response (CR) as Responder (R) and patients with Progressive Disease (PD) as non-responders (NR). *P* values were determined by performing the Fisher’s Exact test on a contingency table with columns indicating clinical benefit and rows indicating the signature levels.

### Statistical analysis

For comparison between matched patients and for intra-individual comparison (different T-cell subsets within the same district), the Friedman test and Wilcoxon U-test were used. For the comparison between unmatched patients, we employed the non-parametric Kruskal–Wallis test and Mann–Whitney U-test. All *P* values were adjusted with Bonferroni correction for multiple comparisons, when appropriate. Correlation analysis was performed with the Spearman and Pearson test. Box graphs show the 25th and 75th percentiles, median values, and outliers. Scatter-plot graphs show median values with interquartile range. A *P* value ≤ 0.05 was considered significant. Significance is represented as *P ≤ 0.05, **P ≤ 0.01, ***P ≤ 0.001, ****P ≤ 0.0001. Statistical evaluation was performed with SPSS 21.0 (SPSS Inc., Chicago, IL, USA) for Windows.

## Results

### RNA-Seq reveals a different transcriptional profile in CD8^+^PD1^+^ Ag-specific T-cell clones depending on CD28 expression

**Fig. 1:**
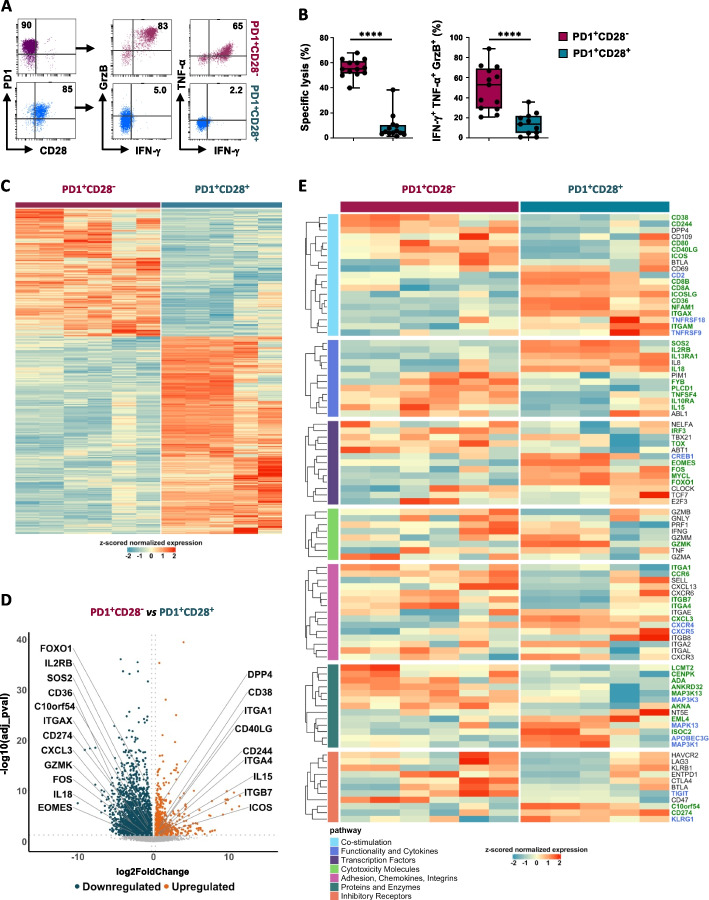
Functional and transcriptional profiles of Ag-specific CD8^+^PD1^+^ T-cell clones are distinctively related to CD28 expression. **A** Representative flow cytometry plots from two Ag-specific CD8^+^ T-cell clones isolated from peripheral blood of melanoma patients, with PD1^+^CD28^−^ (Melan-A-specific clone PT15.26, upper) or PD1^+^CD28^+^ (gp100-specific clone, PT08.24, lower) phenotype. Right, polyfunctionality of the same clones, as evaluated by intracellular staining of GrzB, IFN-γ, and TNF-α following activation with a Melan-A^+^/gp100^+^ melanoma cell-line for 4-5h in the presence of protein transport inhibitors. The percentage of positive expression is shown. **B** Pooled results from Ag-specific CD8^+^ T-cell clones isolated from 5 melanoma patients, grouped for PD1^+^CD28^−^ or PD1^+^CD28^+^ phenotype, in terms of anti-tumor cytotoxicity, as evaluated by Cr^51^ release (left, *n* = 26) and polyfunctionality (right, *n* = 24) against a Melan-A^+^/gp100^+^ melanoma cell-line. Each dot represents the mean value from two to five independent experiments performed on a single T-cell clone. *P* values were calculated by the Mann–Whitney unpaired two-sample test. ****P ≤ 0.0001. **C-F** RNAseq data from CD8^+^ PD1^+^CD28^−^ (*n* = 3) and PD1^+^CD28^+^ (*n* = 3) Ag-specific T-cell clones isolated from 4 melanoma patients. **C** Heatmap showing unsupervised clustering of the total 3700 genes (*P*_adj ≤ 0.05) in the two T-cell subsets. **D** Volcano plot analysis, based on differential expression of genes (DEG) between the two T-cell clone types. Blue dots indicate down-regulated genes (*P*_adj ≤ 0.05, FC < -0.25), and orange dots indicate up-regulated genes (*P*_adj ≤ 0.05, FC > 0.25). Selected genes are reported. The complete list of DEG is reported in Table S3. **E** Heatmap illustrating selected function-associated gene expression. In green, genes with *P*_adj ≤ 0.05; in blue, genes with *P*_adj ≤ 0.1; in black, genes not statistically significant. *P* adjusted values were calculated by the Benjamini & Hochberg adjustment method

By taking advantage of a panel of CD8^+^ Ag-specific (Melan-A^+^ or gp100^+^) T-cell clones isolated from the peripheral blood of melanoma patients, we recently reported that the inhibitory effect of PD1 is associated with the co-expression of CD28 [23, 26, 27], consistent with earlier studies [18]. Indeed, as illustrated in Fig. 1A and B, PD1^+^CD28^−^ T-cell clones show superior functionality compared to their PD1^+^CD28^+^ counterparts, both in terms of specific anti-tumor cytotoxicity (Fig. 1B, left) and concurrent production of lytic molecules (Fig. 1B, right).

To gain deeper information about the molecular features underlying this functional divergence we compared the transcriptional profiles of unstimulated PD1^+^CD28^−^ and PD1^+^CD28^+^ Ag-specific CD8^+^ T-cell clones by RNA-Seq (Fig. 1C-E).

Heatmap shows an unsupervised clustering of the 3700 genes for the two groups of clones (Fig. 1C and Table S3). Volcano Plot analysis, based on differential expression of genes (DEG) (with adjusted *P* ≤ 0.05) shows a total of n = 580 genes, with 54 genes up-regulated and 526 downregulated in CD28^−^ *vs* CD28^+^ T-cell clones (Fig. 1D).

Most transcripts clustered specifically within the two groups (Fig. 1E). More in detail, among co-stimulatory genes, CD28^−^ Ag-specific T-cell clones expressed highest *ICOS, CD40LG, CD80, CD38, CD244 and DPP4* (CD26) while CD28^+^ counterparts showed *CD36 and ITGAX* (CD11c) (Fig. 1D,E). The highest *IL-10RA* and *IL-15* transcription was observed in CD28^−^ T cells, while the highest *IL-2RB, SOS2,* and *IL-18* were found in CD28^+^ Ag-specific T cells. Among integrin/chemokine transcripts, PD1^+^CD28^−^ Ag-specific T cells showed highest *ITGA4* (CD49d, integrin α4), *ITGB7* (integrin ꞵ 7), *ITGA1* (CD49a, integrin α1), and a trend for higher *CXCL13*, while CD28^+^ T cells expressed highest *CXCL3* and a trend for higher *CXCR5* expression (Fig. 1D, E). The central regulator of T-cell exhaustion *TOX* and the Interferon regulatory factor *IRF3* were highest in CD28^−^ T-cell clones (Table S3, Fig. 1E), while at variance, *FOXO1, FOS,* and *EOMES* were more expressed by CD28^+^ Ag-specific T cells (Fig. 1D, Table S1). Expression of *TCF7* mRNA was independent of the presence of CD28 in this setting. Transcripts coding for activation molecules were independent of CD28 expression, according to their unstimulated state, except for the pre-exhausted T-cell effector molecule *GZMK* [28], which was highest in CD28^+^ T cells (Fig. 1D). Among genes coding for inhibitory molecules, *VISTA* and *CD274* (PD-L1) were highest among CD28^+^ T cells (Fig. 1D), while *TIGIT* showed a strong trend of higher expression in the absence of CD28 (Table S3). KLRG1, a marker of both terminal differentiation [29] and context-dependent T-cell activation [28] showed a trend for higher expression in CD28^+^ T cells (Fig. 1E). Taken together, these data reveal that, although poorly functional, Ag-specific CD28^+^ T cells express a mRNA signature associated with a pre-exhausted memory-like pattern.

To test this hypothesis, we conducted a "gene set enrichment analysis" (GSEA), on genes depicted in Fig. 1C-E, employing gene sets associated with pre-exhausted and exhausted T cells from the literature (Fig.S1). While the observed differences did not reach statistical significance, there was a discernible trend in Ag-specific CD28^−^ T-cell clones towards exhibiting a higher expression of an "exhausted" transcriptional signature, while "pre-exhaustion" genes displayed a stronger correlation with CD28^+^ cells (Fig. S1).

The transcriptional analysis presented here corroborates the comprehensive multiparametric analyses we conducted on Ag-specific PD1^+^CD28^−^ and PD1^+^CD28^+^ CD8^+^ T-cell clones in our prior studies [26, 27]. Consistent with their transcriptional profiles, we observed that Ag-specific PD1^+^CD28^−^ T cells express the highest levels of ICOS, directly implicated in their high polyfunctionality, despite concomitant high expression of inhibitory receptors PD1, LAG-3, and TIM-3 [26]. Additionally, the expression of CXCR5, recognized as a distinctive marker of proliferative T cells following PD1 blockade [20], was most pronounced in PD1^+^CD28^+^ T cell clones [27], thereby confirming a strong trend towards higher CXCR5 mRNA expression (Fig. 1E). While both CD122 and NKG2D mRNAs exhibited greater expression in PD1^+^CD28^+^ T-cell clones (Table S3), at the protein level their expression varied heterogeneously among the different clones [28]. Notably, KLRG1, which displayed a tendency towards higher expression at the transcriptional level in PD1^+^CD28^+^ T cell clones, was not consistently detected as a protein in all Ag-specific CD8^+^ T cells [27].

### The lack of CD28 favors the functionality of Ag-experienced CD8^+^PD1^+^ peripheral T cells of cancer patients

**Fig. 2:**
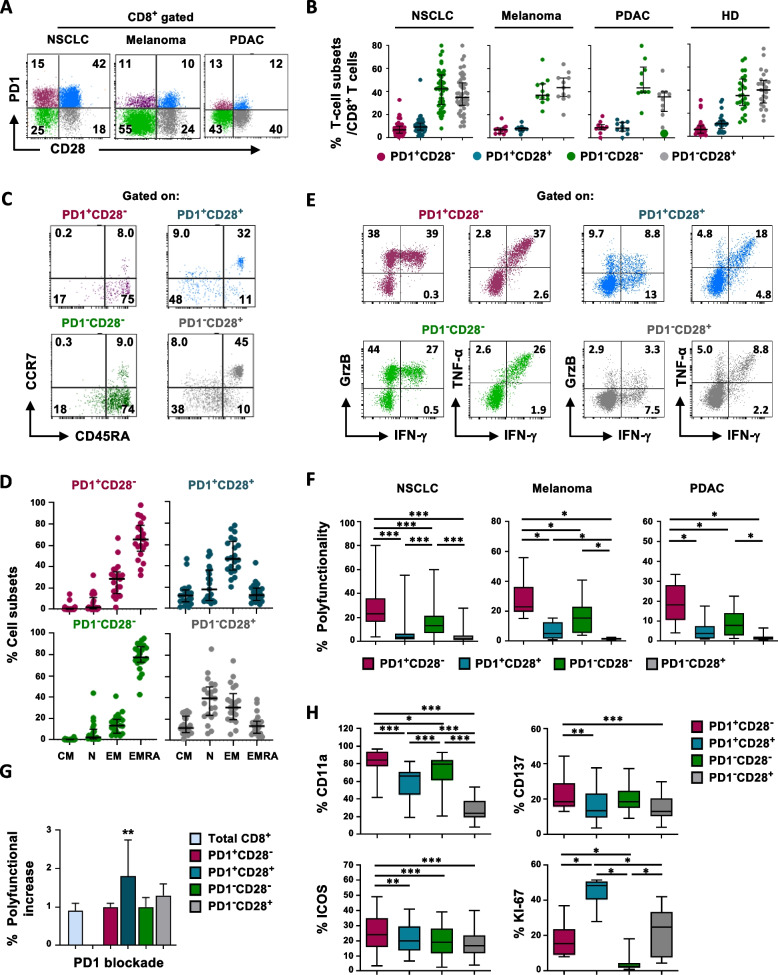
Frequency, differentiation stage and functionality of peripheral CD8^+^ T-cell subsets defined by PD1 and/or CD28 expression. **A** Representative flow cytometry plots showing PD1 *vs* CD28 staining, in ex vivo CD8^+^ T cells from PBMC of patients with three different tumors, as indicated. **B** Percentage of circulating PD1^+^CD28^-^, PD1^+^CD28^+^, PD1^−^CD28^−^ and PD1^−^CD28^+^ subsets within CD8^+^ T cells from NSCLC (*n* = 48), melanoma (*n* = 10), PDAC (*n* = 10) and HDs (*n* = 25). No statistical difference was observed between the proportion of the four subsets in samples of different origins (Kruskal–Wallis). **C** Representative flow cytometry plots showing CCR7 *vs* CD45RA staining, in gated PD1/CD28 T-cell subsets, as indicated. **D** Percentage of CM (CCR7^+^CD45RA^−^), N (CCR7^+^CD45RA^+^), EM (CCR7^−^CD45RA^−^), and EMRA (CCR7^−^CD45RA^+^) within the gated PD1/CD28 T-cell subsets, in NSCLC patients (*n* = 21). **E** Representative flow cytometry plots showing intracellular GrzB, IFN-γ and TNF-α production, within the PD1/CD28 T-cell subsets, following anti-CD3 mAb activation (5-6h) in the presence of protein transport inhibitors. **F** Percentage of polyfunctional cells, defined as the ability to produce two or more lytic molecules simultaneously, within the PD1/CD28 T-cell subsets, in NSCLC (*n* = 35), melanoma (*n* = 10), and PDAC (*n* = 9) patients. **G** Polyfunctional increase following anti-CD3 mAb activation in the presence of anti-PD1 blockade with the specific mAb (*n* = 9), within the PD1/CD28 T-cell subsets (Wilcoxon rank test). Histograms show the polyfunctionality fold increase of each T-cell subset after PD1 blockade with respect to its own baseline functionality. **H** Expression of CD11a^high^ (*n* = 22), CD137 (*n* = 21), ICOS (*n* = 31), and Ki67 (*n* = 10) was evaluated by flow cytometry, within ex vivo unstimulated T cells (CD11a, CD137, ICOS) or after 48h of stimulation with anti-CD3 mAb (Ki-67) in PBMC from NSCLC patients gated on PD1/CD28 T-cell subsets. *P* values were calculated using the Wilcoxon rank test, with Bonferroni correction for multiple comparisons. * *P* ≤ 0.05, **P ≤ 0.01, ***P ≤ 0.001. Plots show median values with interquartile range. Expression percentages are shown within the flow cytometry dot plots

Based on results obtained on Ag-specific CD8^+^ T-cell clones, we then investigated the frequency and fitness of CD8^+^ PD1^+^CD28^−^ and PD1^+^CD28^+^ T cells in the peripheral blood of 68 patients with cancer of different derivation (*n* = 48 NSCLC, Table S1), *n* = 10 melanoma, *n* = 10 pancreas ductal adenocarcinoma cancer (PDAC)) and in 25 healthy donors (HDs), by multi-color flow-cytometry analysis (as outlined in the gating strategy, Fig. S2) (Fig. 2A,B), defined by the expression of CD28 and/or PD1 within CD8^+^ T cells. Each of the four T-cell subsets was detected in all samples, without significant statistical differences between the diverse cancers analyzed, with the PD1^−^CD28^+^ and PD1^−^CD28^−^ subsets being the most represented (Fig. 2B).

To uncover potential distinctions between HDs and patients with various cancer types, we examined the distribution frequencies of the four subsets within memory T cells, excluding the naïve CCR7^+^CD45RA^+^ phenotype. This analysis revealed a heightened prevalence of the circulating PD1^+^CD28^−^ T-cell subset in melanoma patients compared to HDs. Concurrently, within the memory-enriched compartment, we observed a higher frequency of the less differentiated PD1^+^CD28^+^ phenotype and fewer PD1^−^CD28^−^ cells in non-small cell lung cancer (NSCLC) patients when compared to both HDs and other tumor types (Fig. S3A).

The analysis of the T-cell differentiation stage by CCR7 and CD45RA expression in CD8^+^PD1^+^CD28^−^ T cells isolated from peripheral blood of NSCLC patients, showed a predominant terminal-differentiated effector-memory (EMRA, 65.4%) and effector-memory (EM, 27.3%) phenotype (Fig. 2C,D). At variance, PD1^+^CD28^+^ CD8^+^ T cells were more represented within EM T cells (48.9%). PD1^−^CD28^+^ T cells were found mainly among naïve T cells (Fig. 2C,D). Remarkably, a short-term (5h) activation with anti-CD3 mAb transiently increased an EM phenotype at different extents in PD1^+^CD28^−^ (from 27.3% to 46.6%) and PD1^+^CD28^+^ T cells (from 48.9% to 61.5%) (Fig. S3B, middle panels). Differently, a longer anti-CD3 mAb stimulation (48h) reverted the differentiation phenotype to the baseline expression (Fig. S3B, bottom panels).

Interestingly, the CD8^+^PD1^+^CD28^−^ T-cell subset showed the highest polyfunctionality in each melanoma, NSCLC, and PDAC cancer patients (Fig. 2E,F). Considerable polyfunctionality was also displayed by CD8^+^PD1^−^CD28^−^ T cells, confirming their role as functionally competent EMRA effector T cells (Fig. 2F). At variance, both CD8^+^PD1^−^CD28^+^ T cells and PD1^+^CD28^+^ subgroups showed very low polyfunctionality (Fig. 2E,F), in agreement with observations obtained in CD8^+^ Ag-specific T-cell clones (Fig. 1A,B). According to the observation that CD28 is the main down-stream molecular target of PD1 [18], polyfunctionality of the PD1^+^CD28^−^ T-cell subset was not modified by PD1 blockade, while the poor functional fitness of PD1^+^CD28^+^ T cells was improved by anti-PD1 mAb (Fig. 2G).

Next, we focused on the role of co-stimulatory molecules other than CD28, *i.e*. ICOS [30], CD137 (4-1BB) [31], and integrin CD11a [32] (Fig. S2) as contributors to the functional fitness of PD1^+^CD28^−^ T cells in peripheral blood of NSCLC cancer patients. ICOS was expressed to the highest extent among PD1^+^CD28^−^ T cells (Fig. 2H), according to what we have previously observed in Melan A-specific CD8^+^ T cell clones [26]. CD137 and CD11a^high^ were also expressed to the highest extent in the PD1^+^CD28^−^ subset. In agreement with the role attributed to CD11a [33], the less differentiated PD1^−^CD28^+^ subset showed the lowest levels of this Ag-experienced T-cell marker (Fig. 2H). The analysis of Ki-67 (Fig.S2), unveiled the highest proliferative capability for PD1^+^CD28^+^ T cells after 48 h of stimulation with α CD3 mAb (Fig. 2H). However, it is important to note that we cannot rule out that a significant fraction of this population might originate from PD1^−^CD28^+^ki67 ^high^ T cells, which upregulate PD1 following 48h stimulation, as previously reported [27].

These data point out that both PD1^+^CD28^−^ and PD1^+^CD28^+^ T-cell subsets are detectable in *ex-vivo* peripheral blood of patients with cancers of different derivations and resemble functional and RNA-Seq analysis results from Ag-specific T-cell clones. Of relevance, we demonstrate that the absence of CD28 provides a functional advantage to Ag-experienced (ICOS^+^, CD137^+^, CD11a^high^) PD1^+^CD8^+^ T cells in the setting of peripheral blood of cancer patients.

### CD28 expression dictates an opposite functional behavior in CD8^+^PD1^+^ T cells with the transition from the periphery to the tumor site in NSCLC patients

**Fig. 3:**
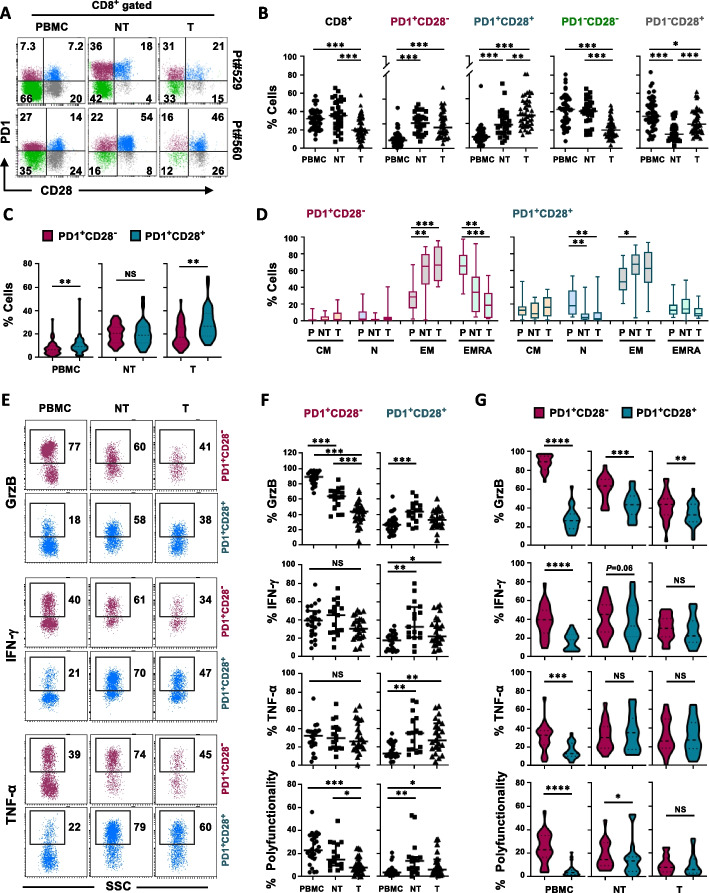
PD1^+^CD28^−^ and PD^+^CD28^+^ T-cell subsets show different functionality in peripheral blood and tumor site. **A** Representative flow cytometry plots showing PD1 *vs* CD28 staining, in ex vivo CD8^+^ T cells isolated from PBMC (P), adjacent non-tumor tissue (NT), and tumor site (T), in two NSCLC patients. **B** Percentage of total CD8^+^, gated according to their dimensions (FSC, forward-scatter and SSC, side-scatter parameters), and PD1/CD28 T-cell subsets, from the periphery to the tumor site, in 68 NSCLC patients (PBMC, *n* = 48; NT, *n* = 30; and Tumor, *n* = 43). *P* values were calculated by unpaired Mann–Whitney test, with Bonferroni correction for multiple comparisons. **C** Comparison between the frequencies of CD8^+^ PD1^+^CD28^−^ and PD1^+^CD28^+^ T cells within the same district (Wilcoxon rank test). **D** Percentage of CM, N, EM, and EMRA, evaluated by CCR7 and CD45RA expression, within CD8^+^ PD1^+^CD28^−^ and PD1^+^CD28^+^ T-cell subsets, in ex vivo unstimulated PBMC, NT and tumor site in NSCLC patients (*n* = 21) (Mann–Whitney test, with Bonferroni correction). **E** Intra-cellular GrzB, IFN-γ, and TNF-α expression following anti-CD3 mAb activation (5-6h) in the presence of protein transport inhibitors, in CD8^+^ PD1^+^CD28^−^ or PD1^+^CD28^+^ T-cell subsets, in ex vivo T cells from PBMC, NT and tumor site, in one NSCLC patient. **F-G** Quantification of single or simultaneous lytic molecule production, in CD8^+^ PD1^+^CD28^−^ or PD1^+^CD28^+^ T-cell subsets, from the periphery to the tumor site (**F**), or in the comparison between the two subsets within the same district (**G**), in 35 NSCLC patients (PBMC, n = 27; NT, *n* = 17; T, *n* = 24). F, Mann–Whitney test, with Bonferroni correction. G, Wilcoxon rank test. *P ≤ 0.05, **P ≤ 0.01, ***P ≤ 0.001, ****P ≤ 0.0001. NS, not significant. Plots show median values with interquartile range. Expression percentages are shown within the flow cytometry dot plots

Focusing on NSCLC, then we evaluated the dynamic distribution and functionality of PD1^+^CD28^−^ and PD1^+^CD28^+^ T-cell subsets in 68 treatment-naïve NSCLC patients (Table S1), by comparing peripheral blood, adjacent non-tumor (NT) tissue and tumor site (T) (*n* = 32 matched NSCLC patients). The transition from the periphery to the NT tissue and the tumor site revealed a different phenotypic distribution of CD8^+^ T cells according to their PD1 and CD28 expression (Fig. 3A-C). While overall CD8^+^ T cells declined, the frequency of both PD1^+^CD28^−^ and PD1^+^CD28^+^ subpopulations increased from the periphery to the NT tissue (8% *vs* 21% and 10% *vs* 20%, respectively) (Fig. 3B), showing similar frequency at this site (Fig. 3C). A gradual rise of PD1^+^CD28^+^ T cells was found from the periphery to the lung tissue, with a further increase from NT to the tumor site (20% *vs* 29.5%) (Fig. 3B). Differently, the PD1^+^CD28^−^ T-cell subset, after a substantial rise at NT, did not further increase in the tumor, where its frequency was significantly lower than the CD28^+^ counterpart (19% *vs* 29.5%, *P* = 0.007) (Fig. 3C). These results clearly demonstrate the advantageous role of CD28 in terms of enhancing migratory capacity from normal tissue to the tumor site. Of note, after anti-CD3 mAb stimulation (48h), a 4 × fold proliferative expansion was observed in PD1^+^CD28^+^ cells derived from peripheral blood (Fig. S4A), revealing an intrinsic expansion capability.

The assessment of the differentiation stage revealed that PD1^+^CD28^−^ T cells were mainly EMRA in the periphery, while showed mostly an EM phenotype in the tumor tissue [34] (Fig. 3D). Differently, the presence of CD28 was associated with the preservation of an EM phenotype throughout all the sites analyzed (Fig. 3D). This may be either due to the occurrence at the tumor site of a CD28^−^CD45RA^−^ T-cell population, which lost CD28 following a persistent Ag-mediated local stimulation or be the consequence of an activation eventually occurred when T cells enter in the tumor, congruous with the observation that EM T cells increase only after short-term activation with anti CD3-mAb (Fig. S3B).

Of note, the presence/absence of CD28 critically affected functionality of PD1^+^ CD8^+^ T cells in the transition from periphery to the lung tissue. In particular, the absence of CD28 was associated with a gradual decline in terms of granzyme-B (GrzB) production from peripheral blood to the NT tissue and the tumor site (Fig. 3E,F). Similar functional impairment was observed for IFN-ɣ, although differences did not reach statistical significance, while TNF-α did not show substantial changes between the three districts analyzed (Fig. 3E,F). However, despite the functional decline, PD1^+^CD28^−^ T cells showed residual functional fitness at the tumor site, as revealed by the slight but significant advantage over PD1^+^CD28^+^ T cells in terms of GrzB production (Fig. 3G). Conversely, the presence of CD28, which was associated with poor polyfunctionality in the periphery, increased the functional capability of PD1^+^ T cells with the passage to the NT and tumor site (Fig. 3F).

In detail, the comparison between the subsets in the different districts showed a clear functional advantage of PD1^+^CD28^−^ T cells over the PD1^+^CD28^+^ subset either in terms of GrzB (*P* < 0.0001), IFN-ɣ (*P* < 0.0001), TNF-α (*P* = 0.001) and polyfunctionality (*P* < 0.0001) (Fig. 3G) in the periphery. Differently, differences between the two subsets were clearly less evident at the NT and even not significant in the tumor site, either in terms of IFN-ɣ, TNF-α and polyfunctionality, with a partial functional advantage of PD1^+^CD28^−^ T cells only in terms of GrzB (*P* < 0.003) (Fig. 3G).

We then analyzed the frequencies and functionality of PD1^high^ and PD1^low^ expression among the subsets characterized by the presence/absence of CD28 (Fig. S4B). In both CD28^−^ and CD28^+^ T cells, PD1^high^ frequency increased significantly in NT and further raised at the tumor site. Remarkably, at the tumor site we found that high level of PD1 selectively impairs functionality in terms of IFN-γ production only in the absence of CD28 (Fig. S4C). We also noted a pronounced and gradual decline in GrzB production in CD28^-^ T cells, observed from peripheral blood to normal tissue and the tumor site. Notably, this impairment was independent of PD1 expression levels (Fig. S4C).

Collectively these data indicate that, although overall CD8^+^ PD1^+^ T cells may encounter unfavorable conditions in the tumor microenvironment, a significant functional response may still occur, likely regulated by distinctive mechanisms depending on the presence/absence of CD28.

### ICOS advantages functionality of CD8^+^PD1^+^CD28^−^ T cells in the periphery but not at the tumor site

**Fig. 4:**
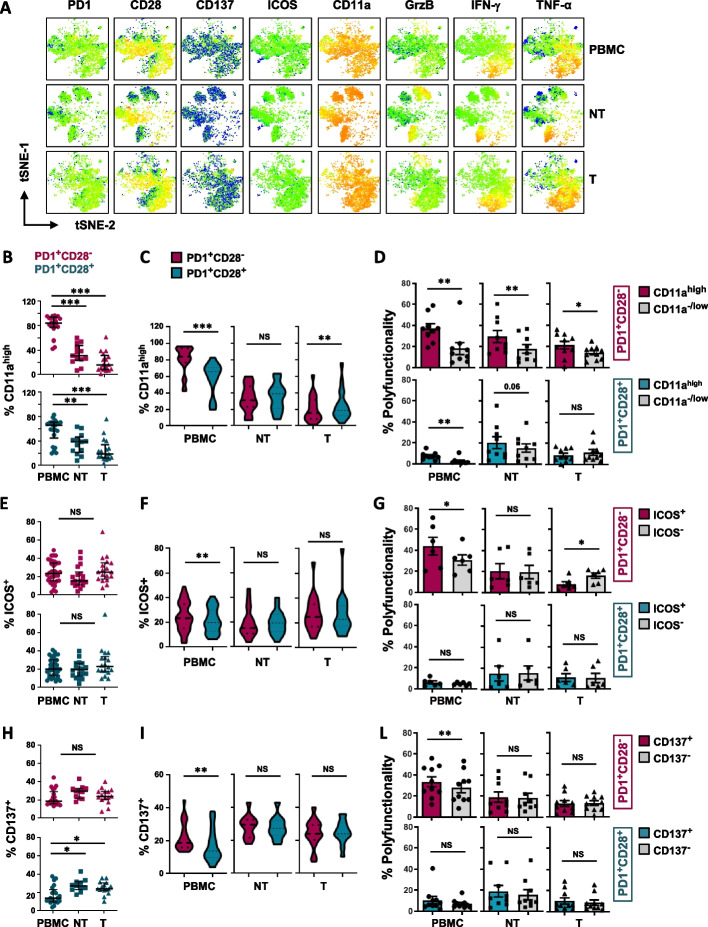
Functional benefit provided by CD11a^high^, ICOS and CD137 depends on the PD1/CD28 phenotype and localization. **A** Expression levels of co-stimulatory receptors and lytic molecules in T cells from PBMC, adjacent non-tumor tissue (NT), and tumor site (T), in a representative NSCLC patient, illustrated by t-SNE plots. **B-E–H** Expression of CD11a^high^ (B, *n* = 23), ICOS (E, *n* = 21) and CD137 (H, *n* = 38), as evaluated by multiparametric flow cytometry, within ex vivo unstimulated *T* cells gated on CD8^+^ PD1^+^CD28^−^ and PD1^+^CD28^+^ subsets, from the periphery to the tumor site of NSCLC patients (Mann–Whitney test, with Bonferroni correction for multiple comparisons). **C-F-I** Expression of CD11a^high^ (C), ICOS (F), and CD137 (I), in the comparison between CD8^+^ PD1^+^CD28^−^ and PD1^+^CD28^+^ T cells within the same district (Wilcoxon rank test). **D-G-L** Polyfunctional ability in CD8^+^ PD1^+^CD28^−^ or PD1^+^CD28^+^ T-cell subsets following activation with anti-CD3 mAb (5-6h), between gated CD11a^high^ *vs* CD11a^low/−^ (D, *n* = 10), ICOS^+^ *vs* ICOS^−^ (G, n = 6) and CD137^+^ *vs* CD137^−^ (L, *n* = 10) cells, in the different districts of matched patients (Wilcoxon rank test). * *P* ≤ 0.05, **P ≤ 0.01, ***P ≤ 0.001. NS, not significant. Plots show median values with interquartile range

To better identify the subpopulation that could benefit most from the expression of CD11a, ICOS and CD137 (Fig. 4A), we compared the T-cell subsets expressing or lacking the markers in the three matched sites of NSCLC patients.

CD11a^high^ was highest in the CD28^−^ T-cell subset in peripheral blood, however a dramatic decline was observed within both subsets with the transition to the tumor site (Fig. 4B), where the CD28^+^ T-cell subgroup showed highest expression (Fig. 4C). Despite the dramatic CD11a^high^ decrease, its expression was always associated with highest polyfunctionality in PD1^+^CD28^−^ T cells at all sites, while in PD1^+^CD28^+^ T cells it was significantly beneficial only in peripheral blood (Fig. 4D and Fig. S5A).

Expression frequency of ICOS did not show substantial changes within both subsets among the three sites analyzed (Fig. 4E). In contrast, CD137 expression exhibited an increase in PD1^+^CD28^+^ T cells with the transition from the periphery to NT and the tumor site (Fig. 4H).

However, both co-receptors were highest among CD28^−^ T cells in peripheral blood, as described above, while no significant difference was found between the two subsets both at NT and tumor sites (Fig. 4F and I). Remarkably, in PD1^+^CD28^−^ CD8^+^ T cells, ICOS was associated with highest polyfunctionality in periphery but was not beneficial at the tumor site (Fig. 4G), while it did not provide advantage to the CD28^+^ subgroup in any district (Fig. 4G and Fig. S5B). Similarly, CD137 provided highest polyfunctionality to the CD28^−^ T-cell subset only in the periphery (Fig. 4L and Fig. S5C).

Then, to produce evidence that the PD1^+^CD28^−^ T-cell subset may represent a highly functional Ag-experienced circulating population, we analyzed CMV-specific CD8^+^ T cells from peripheral blood of HDs by dextramer staining (Fig. S6). The PD1^+^CD28^−^ phenotype was more represented within CMV^+^ T cells (Fig. S6A,B). Notably, CD11a was more expressed in CMV-specific PD1^+^CD28^−^ T cells, compared to both total CMV^+^CD8^+^ and CMV^+^PD^+^CD28^+^ T cells (Fig. S6C, left). CD137 was significantly higher within CMV^+^CD8^+^ than in total CD8^+^ T cells (Fig. S6C, right). Looking at polyfunctionality among CMV^+^ T cells, the PD1^+^CD28^−^ subset showed greater fitness than the CD28^+^ counterpart (Fig. S6D, left). Of note, the presence of both CD11a and CD137 provided the greatest polyfunctionality to CMV^+^ CD8^+^ T cells only in the absence of CD28 (Fig. S6D, middle and right panels).

In summary, the PD1^+^CD28^−^ CD11a^high^ T-cell subset represents a highly differentiated, functional, and Ag-experienced population circulating in peripheral blood, which declines in the tumor, where the expression of CD11a still favors its functionality. Conversely, CD137 provides optimal co-stimulation to the PD1^+^CD28^−^ subset, both as a whole and Ag-experienced subgroup, only in the periphery. Notably, we revealed that in CD28^−^ T cells, ICOS plays conflicting roles in the periphery relative to the tumor site. Overall, these results suggest that in the absence of CD28, CD11a, ICOS, and CD137 exert different functional roles in the different districts.

### PD1, TIGIT, TIM-3, CTLA-4 and LAG-3 co-expression differently impacts functionality of CD8^+^ T cells depending on CD28 expression and district localization

Tumor-associated acquisition of T-cell dysfunction has been related to increased co-expression of several inhibitory receptors (IRs) including PD1, cytotoxic T lymphocyte antigen 4 (CTLA-4), T-cell immunoglobulin domain and mucin domain-3 (TIM-3), lymphocyte activation gene 3 (LAG-3), and T-cell immunoreceptor with Ig and ITIM domains (TIGIT). However, the functional role of distinctive IR co-expression in CD8^+^ T-cell subsets, in relation to their inter-compartmental distribution, requires further investigation to better define the interplay between these receptors.

We analyzed CD8^+^ T lymphocytes from the three districts of NSCLC cancer patients (n = 19), for single or simultaneous IR expression (gating strategy Fig. S2). Among total CD8^+^ T cells, high TIGIT and TIM-3 and minor levels of PD1, CTLA-4, and LAG-3 were observed in the periphery (Fig. S7A). A significant gradual increase in terms of PD1, TIGIT, and CTLA-4 expression, as evaluated singularly (Fig. S7A) or in co-expression (Fig. S7B), was observed with the transition to NT and tumor site. Notably, except for LAG-3, a positive correlation was found between the expression of all IRs in the periphery and the tumor site (Fig. S7C).

**Fig. 5:**
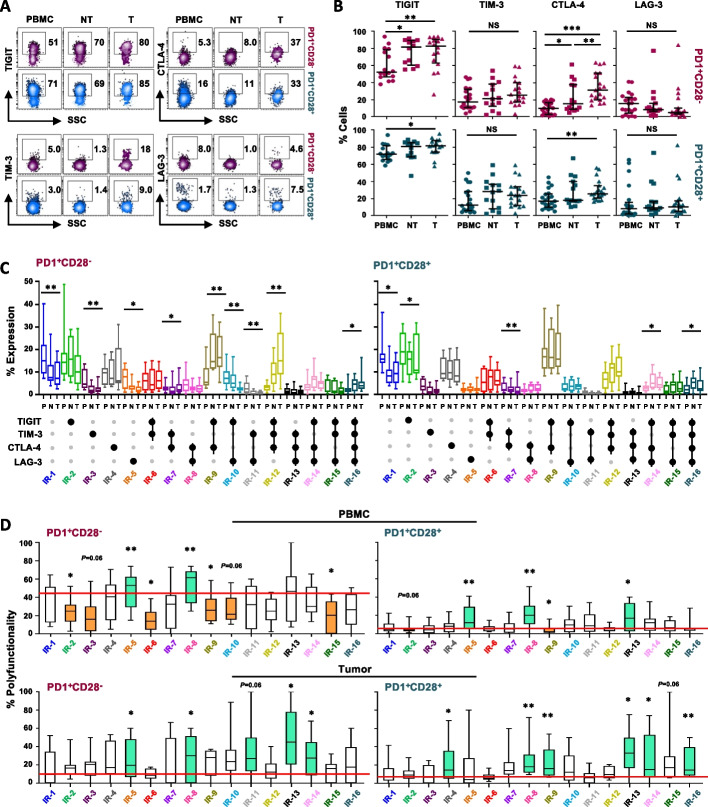
PD1, TIGIT, TIM-3, CTLA-4, and LAG-3 co-expression differently impacts the functionality of CD8^+^ T cells depending on CD28 expression. **A** Representative flow cytometry plots showing TIGIT, CTLA-4, TIM-3, and LAG-3 expression in gated CD8^+^ PD1^+^CD28^−^ or PD1^+^CD28^+^ T-cell subsets, in ex vivo unstimulated cells from PBMC (P), adjacent non-tumor tissue (NT) and tumor site (T) of one NSCLC patient. The percentage of positive expression is shown. **B** Analysis of single TIGIT, CTLA-4, TIM-3, and LAG-3 IR expression in ex vivo unstimulated CD8^+^ PD1^+^CD28^−^ or PD1^+^CD28^+^ T-cell subsets from matched PBMC, NT, and tumor site from 19 NSCLC patients. *P* values were calculated using the Wilcoxon rank test, with Bonferroni correction for multiple comparisons. **C** Quantification of the 16 possible combinations of IRs co-expression, in gated PD1^+^CD28^−^ or PD1^+^CD28^+^ T cells from PBMC, NT, and tumor site from 12 NSCLC patients. Small grey dots show the absence while large black dots indicate the presence of the corresponding inhibitory receptors. *P* values were calculated using the Friedman test between the three districts. **D** Polyfunctionality, evaluated after stimulation with anti-CD3 mAb (5-6h), of the 16 IR subgroups identified within PD1^+^CD28^−^ or PD1^+^CD28^+^ subsets, in cells from peripheral blood and tumor site from 10 NSCLC patients. *P* values were calculated by comparing the polyfunctionality of each different subgroup (IR2-IR16) *vs* the quadruple-negative (IR1) group (orange, lower functionality; green, higher functionality) (Wilcoxon rank test). * *P* ≤ 0.05, **P ≤ 0.01. NS, not significant. Plots show median values with interquartile range

As a next step, we analyzed the pattern of IR expression in PD1^+^CD28^−^ and PD1^+^CD28^+^ T-cell subsets and the impact on their functionality. Only the expression of TIGIT and CTLA-4 increased in both subsets at the tumor site (Fig. 5A,B). When we compared the expression of distinct IRs within the two subsets, we found that TIGIT was the most expressed in both subsets in all sites but showed a significantly higher expression in CD28^+^ T cells in the periphery (*P* = 0.005) (Fig. S8A). This subset also expressed higher CTLA-4 (both in the periphery and NT) and LAG-3 (at the tumor site) than the CD28^−^ counterpart (Fig. S8A).

When we measured the simultaneous IR expression in PD1^+^ T cells with or without CD28, we observed a significant decline of cells lacking all four IRs (quadruple negative, QN) (IR-1) and an increase in terms of cells co-expressing the 4 IRs (quadruple positive, QP) (IR-16), with the transition from periphery to the tumor in both the subsets (Fig. 5C). Double positive (TIGIT^+^CTLA-4^+^) (IR-9) and triple positive (TIGIT^+^CTLA-4^+^TIM-3^+^) (IR-12) were highest among PD1^+^CD28^+^ T cells in periphery and did not increase in the tumor (Fig. 5C). Differently, in the absence of CD28, the low frequencies of double and triple positive IRs gradually increased in the transition to the tumor.

We then analyzed the polyfunctionality of these different subgroups, as compared with their QN counterpart (IR-1), in matched periphery and tumor site (Fig. 5D and Fig. S8B,C). Notably, in the periphery, we observed that TIGIT, expressed alone (IR-2) or in co-expression with CTLA-4 (IR-9) negatively impacted both subsets, while either single TIM3^+^ (IR-3) or double positive (TIGIT^+^TIM3^+^, IR-6) profile impaired only CD28^−^ T-cell functionality (Fig. 5D, orange boxes). At the tumor site, the functionality of both subsets was not impacted by either single or combined IR expression. While the triple positive phenotype (TIGIT^+^CTLA-4^+^TIM-3^+^) (IR-12) did exhibit a gradual and significant increase from the periphery to the tumor site in the absence of CD28, it was not associated with a significant reduction in functionality.

Rather, it was the absence of TIGIT, TIM-3, and CTLA-4, either alone (IR-13 and IR-14) or in combination (IR-5 and IR-8) that improved polyfunctionality of CD28^−^ T cells over QN controls (IR-1) (Fig. 5D, green boxes). PD1^+^CD28^+^ T cells were more functional in the absence of TIGIT, alone (IR-13) or without either TIM-3 (IR-8, and IR-14) or LAG-3 (IR-4 and IR-9) (Fig. 5D, green boxes). Oddly, in the presence of CD28, QP PD1^+^ T cells (IR-16) showed higher functionality as compared with the QN population, a finding that deserves further investigation.

Finally, in the tumor we observed a significant functional decline of PD1^+^ T cells lacking both CD28 and the other four IRs (Fig. S8B,C IR-1), suggesting that in the tumor site, in the absence of CD28, additional mechanisms rather than IR expression restrain T-cell functionality.

### The role of CD28 in the phenotypic change of CD8^+^PD1^+^CD103^+^ T cells from the periphery to the tumor

Considering the critical role of T_RM_ cells which exert a frontline defense against infections and contribute to anti-tumor immune response, we analyzed their frequency within total CD8^+^ T cells and the PD1/CD28 subsets, in the different districts.

**Fig. 6:**
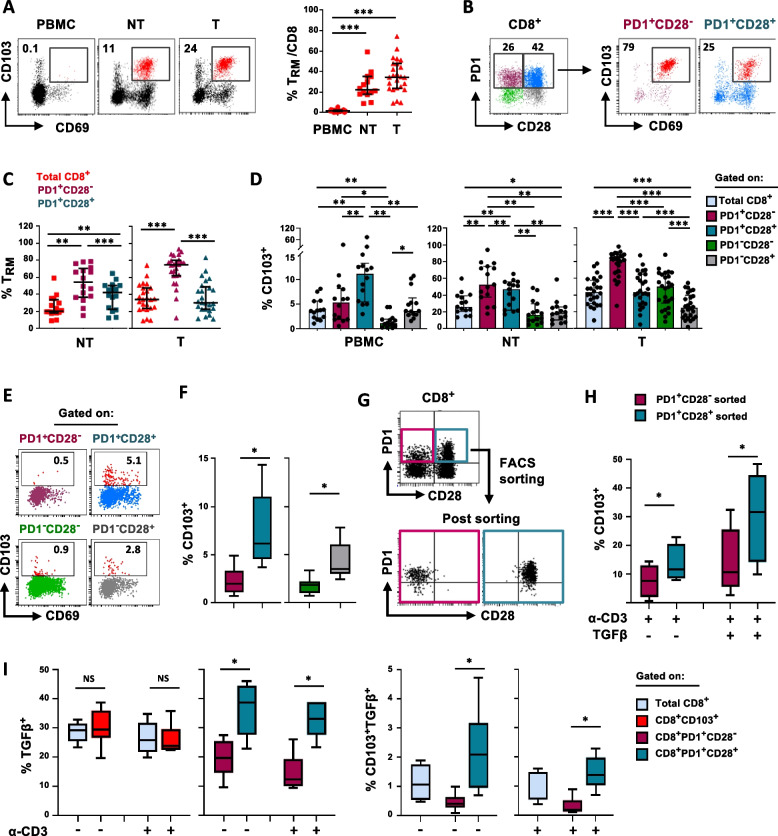
TGFβ-producing T_RM_ precursors are found mainly within circulating CD8^+^ PD1^+^CD28^+^ T cells. **A** Left, representative flow cytometry dot-plots showing CD103 *versus* CD69 staining, in ex vivo CD8^+^ T cells isolated from PBMC, adjacent non-tumor tissue (NT) and tumor site (T), in one NSCLC patient. Right, quantification of ex vivo CD8^+^ T_RM_ (CD103^+^CD69^+^) cells in 25 NSCLC patients (PBMC, *n* = 19; NT, *n* = 14; T, n = 25) (Mann–Whitney test with Bonferroni correction for multiple comparisons). **B** Representative flow cytometry dot-plots showing the proportion of T_RM_ cells in gated CD8^+^ PD1^+^CD28^−^ or PD1^+^CD28^+^ T-cell subsets, in tumor sites. **C** Quantification of T_RM_ cells in total CD8^+^ or in gated PD1^+^CD28^-^ or PD1^+^CD28^+^ cells, in NT (*n* = 14) and tumor sites (*n* = 25) (Wilcoxon rank test with Bonferroni correction). **D** Percentage of CD103 expression in the different T-cell subsets from ex vivo PBMC (*n* = 15), NT (*n* = 15), and tumor site (*n* = 26) of NSCLC patients (Wilcoxon rank test, with Bonferroni correction for multiple comparisons). **E** Representative flow cytometry dot-plots showing CD103 *versus* CD69 staining, in circulating PD1/CD28 T cells from one HD.** F** Percentage of CD103 expression in PD1/CD28 T-cell subsets from HDs (*n* = 9) (Wilcoxon rank test). **G** Representative flow cytometry dot-plots showing PD1 *vs* CD28 staining, before and after FACS-sorting, in one HD. **H** Expression of CD103 in sorted PD1^+^CD28^−^ or PD1^+^CD28^+^ T cells, following 6-days of in vitro expansion by stimulation with anti-CD3 mAb, in the presence or absence of TGFβ (Wilcoxon rank test). **I** Intracellular TGFβ production, evaluated by flow cytometry, in the different T-cell subsets as indicated, with or without anti-CD3 mAb activation (5h), in 6 HDs (Wilcoxon rank test). * *P* ≤ 0.05, **P ≤ 0.01, ***P ≤ 0.001. NS, not significant. Plots show median values with interquartile range

The CD8^+^ CD103^+^CD69^+^ T_RM_ subset was represented within both NT and tumor sites, while found at low frequency or absent in CD8^+^ T cells isolated from the periphery (Fig. 6A). In the NT tissue, the T_RM_ phenotype was higher in both PD1^+^CD28^−^ and PD1^+^CD28^+^ subsets as compared with total CD8^+^ T cells, although enriched in the CD28^−^ T cells (Fig. 6B,C), in line with data of PD1 enrichment in T_RM_ [35]. In the tumor, a considerable increase of T_RM_ phenotype was observed only in CD28^−^ T lymphocytes (percentage of T_RM_ within PD1^+^CD28^−^ T cells, NT *vs* T, *P* = 0.01), strongly indicating that most PD1^+^CD28^−^ T cells infiltrating the NSCLC tumor site possess a T_RM_ phenotype, as previously reported [34, 36].

The existence and identification of circulating T_RM_ progenitors are still controversial matters [12]. We then analyzed the rare circulating T_RM_ precursors and their route from the periphery to the tumor site, by focusing on the analysis of the core T_RM_ cell lineage marker CD103. Although represented at low frequency, higher CD103 expression was found within the PD1^+^CD28^+^ T-cell subset as compared with either PD1^+^CD28^−^ T cells and the two PD1-negative subsets (Fig. 6D, left panel), differently from the scenario identified in the lung tissue, where PD1^+^CD28^−^ T cells showed a gradual increase in CD103 expression, with highest levels at the tumor site (Fig. 6D, middle and right panels).

Based on these observations, firstly, we confirmed the presence of a circulating CD8^+^PD1^+^CD28^+^ T-cell subset showing the highest CD103 expression also in peripheral blood of HDs (Fig. 6E,F). Then PD1^+^CD28^−^ and PD1^+^CD28^+^ subsets from 5 HDs were sorted (Fig. 6G) and cultured in the presence of anti-CD3 mAb, with or without exogenous TGFβ, a well-established CD103 inducer in CD8^+^ T cells [13, 14]. As expected, TGFβ significantly upregulated CD103 expression compared to the baseline stimulated with anti-CD3 mAb in both subsets. Notably, the increase was more pronounced in PD1^+^CD28^+^ cells (Fig. 6H, up-regulation of CD103 in PD1^+^CD28^−^ and PD1^+^CD28^+^ T-cell subsets: 1.4 *vs* 2.76-fold increase respectively).

We then evaluated whether an autocrine TGFβ production occurred in this population. Of note, the PD1^+^CD28^+^ T-cell subset showed the highest TGFβ expression, either unstimulated or activated with anti-CD3 mAb (5h) (Fig. 6I). These data evidence a CD28-associated gradual phenotypic change of CD103^+^PD1^+^ CD8^+^ T cells from the periphery, where are mainly CD28^+^ to the tumor where are mostly enriched of a CD28^−^ phenotype. It is also conceivable that the elevated levels of TGFβ produced by PD1^+^CD28^+^ T cells may potentially contribute to the increased upregulation of CD103 observed in this subset in peripheral blood.

Since T_RM_ express different chemokines to recruit immune cells in the tissues, we then analyzed the crucial player CXCL13 [37]. We detected *ex-vivo* CXCL13 production mainly in CD8^+^ T cells from the tumor site, significantly highest in PD1^+^CD28^−^ T_RM_ (Fig. S9A, upper panels). Notably, after in-vitro expansion in the presence of exogenous TGFβ (6 days), a considerable CXCL13 production was found only in cells from the tumor, mainly characterized by a PD1^+^CD28^−^ T_RM_ phenotype, and not in NT tissue (Fig. S9A-C). These results suggest that distinctive T_RM_ programs may occur in lung tissue and the tumor site, due to environmental variations likely driven by hypoxia, which has been reported to impact both, the heterogeneity, and function of T_RM_.

The CXCL13/CXCR5 axis is crucial for intra-tumor T-cell migration, and accordingly, tumors with high CXCL13 expression exhibit an increased infiltration of activated CD8^+^ CXCR5^+^ T cells [38]. In our setting, we found the highest CXCR5 in CD8^-^ T cells in all districts, in line with reported results [39], while among CD8^+^ T cells it was more represented among the PD1^+^CD28^+^ subset (Fig. S9D and S2). Notably, CXCR5 frequency increased in all subsets with the transition to the tumor, maintaining the highest expression in PD1^+^CD28^+^ cells.

Collectively, these results indicate that highly CXCL13-producing T_RM_ cells lack CD28, while CXCR5-expressing T cells are mainly CD28^+^, suggesting a crucial role of CD28 in establishing this axis.

### CD28 impacts the periphery/tissue compartmentalization, the transcriptional profile and the (dys)functional states of CD8^+^PD1^+^ T cells in NSCLC patients

**Fig. 7:**
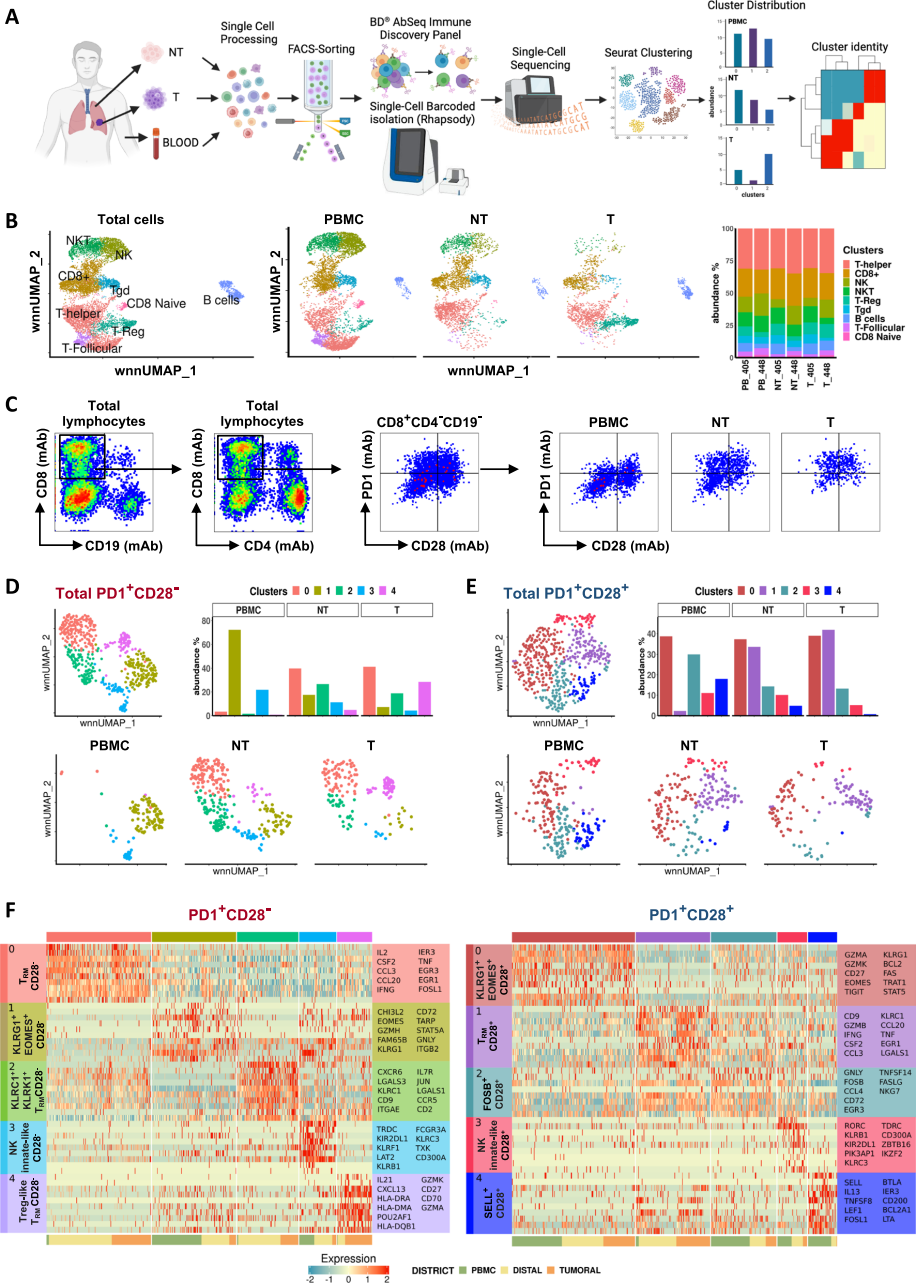
Single-cell RNA-Seq from matched PBMC, NT, and T sites of NSCLC patients identifies different PD1/CD28-associated clusters. **A** Flowchart of the overall study design. Lymphocytes from two NSCLC patients were purified from three matched districts using FACS-sorting and employed for single-cell RNA-Seq, performed with the BD Rhapsody platform. **B** UMAP projection showing the main immune subsets within total cells (*n* = 10,944 cells, right panel) and in the three different districts (middle panels), color-coded according to the cell type. Right, barplot showing the distribution of immune cell types within the different districts in the two patients. **C** Gating strategy to select PD1^+^CD28^−^ and PD1^+^CD28^+^ within a CD8^+^CD4^−^CD19^−^ T-cell population on merged patients. **D-E** UMAP projection of concatenated (upper left panel) or differently distributed cells in the three districts (bottom panels), gated on PD1^+^CD28^−^ (**D**) (*n* = 558) or PD1^+^CD28^+^ (**E**) (*n* = 541) cells, showing the identification of five color-coded clusters for each PD1/CD28 subset. Upper right, Frequency of each cluster across the three districts. The two patients have been grouped together. **F** Heatmap from single-cell analysis showing gene expression levels in cells distributed into clusters (indicated on the top by the same color as in D-E). The top ten marker genes, differentially expressed for each cluster (listed by fold change), are shown on the y-axis. The complete list of DEG is reported in Table S4 and in Table S5. Panel A was created with Biorender

To better explore at single cell level data obtained with multiparametric flow cytometry studies above reported, we extended our analysis by using a targeted multi-omic scRNA-Seq technology (Fig. 7A), able to measure the expression of selected immune-related proteins combined with DEG at the single-cell level, in three different districts of two NSCLC patients. Unsupervised clustering to characterize the overall immune cell profile revealed 10 clusters representative of the main immune cell populations (Fig. 7B). Since both frequency and distribution of the CD8^+^ T cell clusters were comparable between the two patients, the analysis was performed on pooled samples.

Next, we extended our integrated analysis to the CD8^+^ subsets characterized by PD1 and CD28 expression, manually selected based on the specific mAbs, as shown in Fig. 7C. Both PD1^+^CD28^−^ and PD1^+^CD28^+^ T-cell subsets clustered into 5 different groups characterized by distinctive transcriptional profiles and heterogeneously represented within the different districts (Fig. 7D-F and Table S4, S5).

Cluster0 of CD28^−^ and cluster1 of CD28^+^ T-cell subsets (which we named T_RM_CD28^−^ and T_RM_CD28^+^ respectively), were both poorly represented in the periphery while abundant in the NT lung tissue and tumor site. These clusters showed partially overlapping features, characterized by up-regulated transcripts for chemokines *CCL3*, *CCL4* and *CCL20*, *IER3,* and *TNFSF14* among others (Fig. 7F and Table S4, S5) and a functional signature characterized by IFN-ɣ and TNF-α. T_RM_CD28^−^ distinctively showed high *CD69* and *IL-2* transcripts (Fig. 7F and Table S4), high CD103 protein (Fig. S10A,B), but no DEG of the relative *ITGAE* gene. T_RM_CD28^+^ was specifically characterized by the expression of cytotoxic *GRZB* (Table S5), along with *IL12RB2* and *KLRC1* (coding for NKG2A), both related a T_RM_ phenotype. We also identified features of T_RM_ cells in cluster2 of CD28^−^ T cells (that we defined KLRC1^+^KLRK1^+^T_RM_ CD28^−^), including transcripts for *KLRK1* (NKG2D), *ITGAE*, the T_RM_-associated factor *IRF4,* chemokine receptors *CCR5* and *CXCR6*, *CD44* and *JUN*, the latter reported as a molecular determinant of T-cell residency [40] (Table S4). T_RM_ marker *KLRC1* and the immunosuppressive *LGALS1* [41] were shared with T_RM_CD28^+^. KLRC1^+^KLRK1^+^T_RM_CD28^−^ cluster did not show significantly up-regulated cytotoxic-related genes, rather *BTG1,* homeostatic *IL7R*, *IL2RB* and *IL2RA*. T_RM_ determinant CD103 was also found in cluster4 of CD28^−^ T cells, poorly represented within peripheral blood and at the NT tissue, while abundant at the tumor site. The cluster-4, that we named Treg-like T_RM_ CD28^−^, was also characterized by *CXCL13*, *LAG-3*, *TIGIT*, *CTLA-4*, *CD27* transcripts [42] *TNFRSF9* [43] *CD74*, *HLA-DRA* [44], *HLA-DMA* and *HLA-DPA1*. This CD28^−^ signature suggests an effector-Treg-like T_RM_ phenotype, supported by the high overall expression of *ICOS* transcript (Fig. S10C) [43]. In accordance with reported observations [15, 16], our scRNA-Seq data indicate that CD103^+^ T_RM_ cells show a heterogeneous phenotype and distinctive functional states.

Cluster1 of CD28^−^ T cells (KLRG1^+^EOMES^+^CD28^−^), highly represented in the periphery and under-represented at the tumor site, showed transcriptional features of long-lived memory T cells with effector potential, partially shared with cluster0 of the CD28^+^ T-cell subset (KLRG1^+^EOMES^+^CD28^+^) (Fig. 7F and Table S4, S5). The clusters shared up-regulated transcripts for KLRG1, EOMES, T-cell transcription factor STAT5, APOBEC3G, HLA-A, and CST7. KLRG1^+^EOMES^+^CD28^−^ showed a more differentiated cytotoxic profile, with increased *GZMH*, *GNLY,* and *NKG7* transcripts. At variance, KLRG1^+^EOMES^+^CD28^+^ showed, among others, increased *GZMK*, indicative of a long-lived progenitor effector memory phenotype [28, 45], *GZMA*, *CD74*, *TRAT1*, *LCK*, *HLA-DMA* and *HLA-DPA1*. Of note, the CD28^+^ cluster was detected also in the tumor, and accordingly it was enriched by *ITGA4* (CD49), *CCR5* and *CD44*, all contributing to CD8^+^ T-cell infiltrative capacity. Therefore, we can envision that CD28 may dictate the migration of long-lived memory effector CD8^+^ T cells to the tumor site.

Cluster3 of both subsets (which we refer to as NK-innate-likeCD28^− ^and CD28^+^, respectively), represented in peripheral blood and reduced at the tumor site, were characterized by the expression of NK-like markers *KLRB1*, *KLRC3,* and *KIR2DL1*, along with *IKZF2* and *ZBTB16* (PLZF). Notably, NK-innate-likeCD28^−^ cluster expressed additional innate-like associated genes, including *CD244* and *PRDM1*, *RUNX3,* and the cytotoxic *PRF1* (Perforin-1), suggesting the occurrence of a terminally differentiated cytotoxic NK-like T-cell subgroup.

Looking at CD28^+^ T cells (Fig. 7F, right), cluster2 (FOSB^+^CD28^+^), with high expression in peripheral blood and a consistent decline in the tumor, lacked a distinctive transcriptional signature. Cluster4 (SELL^+^CD28^+^) was found almost exclusively in peripheral blood, strongly declined in the NT and was absent at the tumor site. Accordingly, it was characterized by upregulated transcripts for CD62L, IL-7R, the stem-like T cell regulator LEF-1 and Bcl-2. These features likely suggest a subset recirculating through secondary lymphoid organs. Of note, the last two clusters described were found only within PD1^+^CD28^+^ T cells.

### Gene expression signatures identified by scRNA-Seq stratify survival of lung ADC patients and ICB response in advanced NSCLC

We then interrogated the TCGA patient dataset to gain insight about the prognostic potential of the gene expression profiles of three selected clusters in terms of overall survival (OS), by combining extrapolated signatures according to *P* = 0.001 and FC > 1.5, with related transcripts from the "adaptive immunity” pathway of Reactome database. Moreover, to explore the validations of our findings in clinical settings we analyzed data from OAK clinical trials using atezolizumab in patients with metastatic NSCLC.

**Fig. 8:**
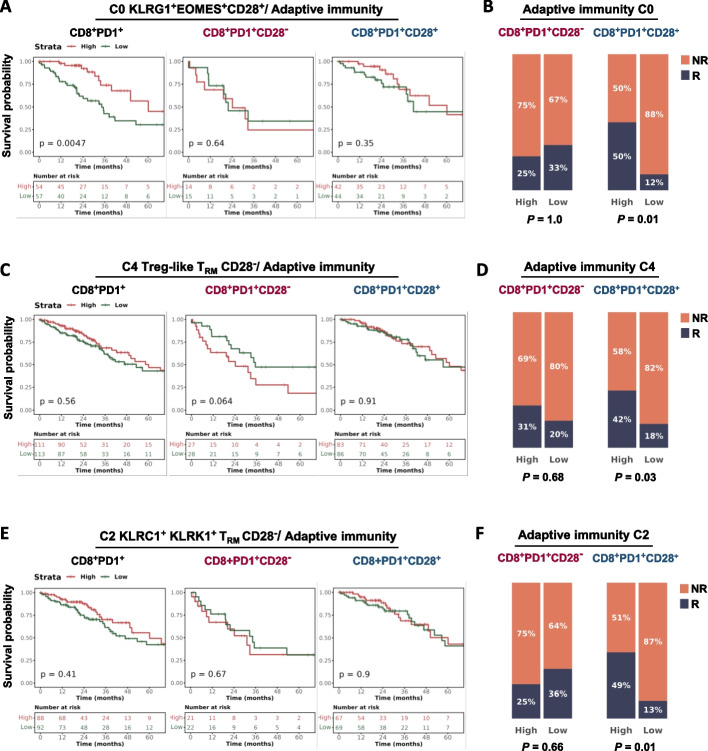
CD28^±^ cluster-derived signatures are predictive of response to PD-L1 blockade in advanced NSCLC patients. **A**,** C, E** Kaplan–Meier curves depicting the OS probability of adenocarcinoma NSCLC patients from TCGA stratified based on High/Low expression levels of C0 KLRG1^+^EOMES^+^CD28^+^/Adaptive Immunity signature (A), C4 Treg-like T_RM_ CD28^−^ /Adaptive Immunity signature (C) and C2 KLRC1^+^KLRK1^+^T_RM_ CD28^-^ /Adaptive Immunity signature (E). For each signature, three TCGA patient cohorts based on the expression levels of CD8, PD1 and CD28 genes are shown: CD8^+^PD1^+^ (left panels), CD8^+^PD1^+^CD28^−^ (middle panels), CD8^+^PD1^+^CD28^+^ (right panels). Number of patients at risk and *P* values are shown. Significance was calculated using the log-rank test. **B**,** D**,** F** Barplots showing the percentage of Responder (R) and Non-Responder (NR) patients in two cohorts of the OAK dataset (PD1^+^CD28^−^, left panels; PD1^+^CD28^+^, right panels), stratified for High/Low scores of Adaptive Immunity/C0 signature (n = 14 and n = 37 for PD1^+^CD28^−^ and PD1^+^CD28^+^, respectively) (B), Adaptive Immunity/C4 signature (*n* = 31 and *n* = 72 for PD1^+^CD28^−^ and PD1^+^CD28^+^, respectively) (D) and Adaptive Immunity/C2 signature (*n* = 23 and *n* = 58 for PD1^+^CD28^−^ and PD1^+^CD28^+^, respectively) (F). *P* values are shown. Significance was computed using the Fisher’s Exact test

We analyzed cluster0 KLRG1^+^EOMES^+^CD28^+^ (Fig. 7E,F and Table S5), considering its occurrence in both peripheral blood and tumor site of NSCLC patients, and found a related signature (C0 KLRG1^+^EOMES^+^CD28^+^/Adaptive Immunity), including 11 genes (CD74, PTPRC, ITGA4, KLRG1, CD8A, TRAT1, CD8B, LCK, CD3E, HLA-DMA, HLA-DPA1), associated with improved OS in TCGA-LUAD patients with CD8^+^PD1^+^ T-cell enriched tumors (*P* = 0.0047) (Fig. 8A). However, this result does not imply a significant impact of CD28 in terms of OS in this setting. By interrogating the OAK dataset, we found that most non-responder patients were significantly associated with a low frequency of the KLRG1^+^EOMES^+^ signature, but this occurred only in PD1^+^CD28^+^ highly infiltrated tumors (*P* = 0.017) (Fig. 8B).

We next focused on cluster4 Treg-like T_RM_ CD28^−^ (Fig. 7D,F and Table S4), due to its potential critical role and its exclusively relevant expression in the tumor, and found a 6 genes signature (C4 Treg-like T_RM_ CD28^−^/ Adaptive Immunity: CD74, LAG3, CD8B, HLA-DMA, HLA-DRA, HLA-DPA1). In the TCGA dataset, although non-significant (*P* = 0.064), low expression of the signature favored patients with CD28^−^ T-cell enriched tumors (Fig. 8C). Differently, this signature in OAK dataset was associated with better response to ICB only when T cells expressed CD28 (*P* = 0.039) (Fig. 8D).

Finally, the signature derived from cluster2 KLRC1^+^ KLRK1^+^T_RM_ CD28^−^ (Fig. 7D,F and Table S4), likely characterized by a T_RM_ phenotype and expressed in both NT and tumor site (C2 KLRC1^+^ KLRK1^+^T_RM_ CD28^−^ /Adaptive immunity) and including KLRC1, CD8A, CD8B, CD3E, KLRK1, was not significantly prognostic in all patient subgroups in the LUAD TCGA dataset (Fig. 8E). At variance, considering the response to the ICB, high levels of the signature were significantly prognostic only in the presence of CD28 (*P* = 0.01) (Fig. 8F).

Altogether these results indicate the clinical value of our signatures and clearly show the different roles of distinctive molecular profiles with respect to prognosis in ADC and prediction of response to ICB.

## Discussion

A deep understanding of the complex network between co-stimulatory and inhibitory receptors is crucial in immune monitoring anti-tumor immune response to identify biomarkers of clinical response in ICB-treated patients.

We and others have shown that the inhibitory effect of PD1 on CD8^+^ T-cell functionality may rely on the co-expression of CD28, which has been proposed as the main target downstream of PD1 [18]. Herein, starting from the analysis of a panel of Ag-specific melanoma CD8^+^PD1^+^ T-cell clones, we have identified different transcriptomic, phenotypic, and functional patterns dictated by the presence/absence of CD28 in peripheral and matched NSCLC tissue.

The highly functional circulating PD1^+^CD28^-^ phenotype previously found within T cell clones [26, 27] was also identified in peripheral blood of patients with different solid tumors, while CD28 did not provide functional advantage to circulating PD1^+^ T cells, either as whole population (Fig. 2F) or Ag-specific lymphocytes (Fig. S6). Indeed, when CD28 is reduced or absent, ICOS may improve CD8^+^ T-cell activation [46], CD137 promotes a proliferative state [47], and CD11a generates high quality synapsis [32] (Fig. 2H and Fig. 4D,G,L).

If this is the landscape in the periphery, in the tumor site PD1^+^CD28^+^ T cells exhibited both frequency enrichment and functional fitness, as recently highlighted likely due to higher cis-B7/CD28 interaction [48], also reminding the ability of myeloid APC niches to generate polyfunctional effector CD8^+^ T cells, specifically associated with CD28 co-stimulation in response to PD1 blockade [21]. Differently, CD28neg T cells showed a gradual polyfunctional decline and a switch from an EMRA to EM phenotype (Fig. 3D), suggesting a possible origin from newer CD28^−^CD45RA^−^, which lost CD28 as a result of Ag-mediated stimulation.

Of note, in the tumor site also CD137 and ICOS failed to provide functional advantage to CD28^-^ T cells, nevertheless still sustained by CD11a^high^, although expressed only in a small fraction of the subset (Fig. 4). Our data are in line with the role of CD137 as a relevant immunosuppressive molecule Treg-associated in the tumor microenvironment, different from what observed in periphery [49], and in agreement with an infiltrating CCR8^+^ICOS^+^ CD8^+^ Treg signature of disease progression found in NSCLC [50]. To highlight this different CD137 and ICOS related scenario found in the tumor with respect to the periphery, when CD28 is lacking, we cannot rule out that a fraction of intra-tumor PD1^+^CD28^−^ T cells may have switched to a Treg-like phenotype, likely driven by the local milieu [51].

Considering the role of CD28 as main target of PD1 signaling, we may envision that this liaison can play a critical role in the different functional outcome of the two subsets at the tumor site. Indeed, we found CD8^+^ PD1^low^ intensity in the periphery, that increased in both subsets in situ, significantly impairing T-cell functionality mostly in the absence of CD28 (Fig. S4C). The superior functional fitness of circulating CD28^-^ T cells may be ascribed to the reported CD28 inactivation occurring also at low PD1 densities, while TCR signaling is impacted mostly by PD1^high^ expression [18].

We may also envision that in the tumor, a potentially higher PD1/PDL1 engagement can occur [52], due to both increased PD1 expression and high PD-L1 availability. This can lead to an enhanced PD-L1/CD80 cis-heterodimerization that, by preserving the ability of CD80 to activate CD28 [53], can sustain the functionality of intra-tumor CD28^+^ T cells.

Considering the impact of other IRs than PD1 on T-cell functional state, a higher co-expression of TIGIT and CTLA-4 could also partially contribute to explaining the overall lower functionality of circulating PD1^+^CD28^+^ T cells. However, CD28^+^ T-cell functional fitness was not improved by the absence of the 4 IRs evaluated, enforcing the role of PD1 alone in impairing the functional competence of CD8^+^ T cells through a CD28-dependent mechanism in peripheral blood of NSCLC patients. At variance, circulating PD1^+^CD28^−^ T cells were mostly affected by TIM-3 and TIGIT, either alone or co-expressed. At the tumor site, functionality was impacted by the absence of distinctive patterns of IRs rather than their presence. Of note, mainly the lack of TIGIT, either alone or without TIM-3 and CTLA-4, was associated with better effector fitness in CD28^−^ T cells (Fig. 5D). Differently, in CD28^+^ T cells, the concomitant lack of CTLA-4, TIGIT, and TIM-3 did not provide a functional advantage, suggesting that CD28 co-stimulation is able to overcome the inhibitory effect of CTLA-4 [54] in the absence of TIGIT and TIM-3. Overall, our results contribute to a deeper insight on the potential impact of presence/absence of CD28 in the context of distinctive patterns of IR expression. Further studies are required to investigate the role of CD226, which works in parallel with CD28 and has been recently reported to be inhibited by TIGIT [55].

The impact of T_RM_ cells in anti-tumor responses has been largely investigated, but the origin of these cells is still unclear, although the existence of a circulating precursor committed to a T_RM_ phenotype within a T effector progeny has been reported [12]. Our finding of a modest circulating population within PD1^+^CD28^+^ T cells, with higher CD103 expression as compared with CD28^−^ T cells and an intrinsic capacity to self-up-regulate autocrine TGFβ (Fig. 6), speaks in favor of a circulating subgroup privileged in terms of both development and maintenance of a T_RM_ phenotype. Considering the critical role of the CXCL13/CXCR5 axis in modulating lymphocyte infiltration [56, 57], our evidence that intra-tumor PD1^+^CD28^−^ T_RM_ possess highest CXCL13 chemokine, while memory stem-like marker CXCR5 [58] is mostly expressed by CD28^+^ (CD103 ^low^) T cells (Fig. S9), favours a scenario of CD28^−^CXCL13^+^ T cells attracting memory reservoir CD28^+^CXCR5^+^ T cells within the tumor site in our NSCLC setting.

At single cell level, each PD1^+^CD28^−^ and PD1^+^CD28^+^ T-cell subset was further dissected into 5 clusters, showing heterogeneous transcriptional profiles, revealing distinctive phenotypes and functional states.

As highlighted by the literature [15, 16], we found heterogeneous T_RM_ cell states (Fig. 7F and Table S4,S5). Although reported mainly as CD28^−^ [59], we found a T_RM_CD28^+^ cluster which we could envision as an earlier T_RM_ population (Fig. 6D-I). A trend of prediction for poor OS was found for the signature derived from cluster4 (Treg-likeT_RM_ CD28^−^), in agreement with reported observations [60]. Notably, these signatures were highly represented in advanced lung cancer patients responding to atezolizumab, only in the presence of CD28 (Fig. 8C-F).

Of note, our cluster-0 KLRG1^+^EOMES^+^CD28^+^ expressed features of plasticity [61] and produced granzyme K [62], compatible with long-lived pre-exhausted effector-memory PD1^+^CD28^+^ T cells [28], possessing a low exhaustion score [4]. In agreement with Guo, we found that a high expression of this signature is associated with better OS in LUAD cohort from TCGA when CD8^+^PD1^+^ T cells were considered, without a direct impact of CD28. These pre-exhausted T cells are more prone to be re-invigorated by ICB and indeed, a high signature was found in responder patients mostly with tumor highly infiltrated by CD28^+^ T cells (Fig. 8A,B). Remarkably, the identified pre-exhausted KLRG1^+^EOMES^+^CD28^+^ cluster was well represented also in periphery, sharing a phenotypical and functional signature with tumor infiltrating T cells, thus likely providing a feasible tool for the identification of responsive patients.

Our results strongly put forward CD28 expression as a key determinant for ICB response in the presence of heterogeneous intra-tumor signatures and different T-cell functional states.

## Conclusions

In conclusion our work highlights how the co-stimulatory molecule CD28 is crucial in dictating different functional states in PD1^+^ CD8^+^ T cells in peripheral blood and tumor site of NSCLC patients. We provided novel signatures and feasible biomarkers able to stratify survival of LUAD patients and predict ICB response in advanced NSCLC.

### Supplementary Information

**Additional file 1: Table S1.** Clinical-pathological characteristics of NSCLC patients.

**Additional file 2:** **Table S2.** Antibodies list.

**Additional file 3: Table S3.** PD1^+^CD28^−^ *vs* PD1^+^CD28^+^ clones DEG.

**Additional file 4:** **Table S4.** PD1^+^CD28^−^ DEG.

**Additional file 5: Table S5.** PD1^+^CD28^+^ DEG.

**Additional file 6: Figure S1.** Gene Set Enrichment Analysis (GSEA) of Exhaustion and Pre-exhaustion Gene Signatures in CD28^-^ *vs* CD28^+^ T-cell clones. GSEA depicting the distribution of Exhaustion and Pre-exhaustion gene signatures (Chu Y et al., Nat Med. doi: 10.1038/s41591-023-02371-y), within the differentially expressed gene list resulting from the CD28^−^ *vs* CD28^+^ clones’ comparison. Genes are ranked based on the DESeq2 Wald test statistic (“stat”). The plots display the running sum of the Enrichment Score (ES) across the gene list (green line) and ES peak (red dashed line). GSEA reveals a clear trend for a higher expression of an “exhausted” transcriptional signature, while “pre-exhaustion” genes were more correlated with CD28^+^ cells.

**Additional file 7:** **Figure S2.** Gating strategy of representative immunophenotyping panels for the staining with mAbs employed in the study. Representative dot-plots from NSCLC patients, as evaluated by multicolor flow cytometry gated on total CD8^+^ T cells and PD1/CD28 subsets, either unstimulated or stimulated with anti-CD3 mAb. FMO, fluorescence minus one control.

**Additional file 8:** **Figure S3.** The T-cell differentiation stage impacts the circulating CD28/PD-1 subset distribution and changes as a function of the activation interval. A Unstimulated ex vivo PBMC from NSCLC (n = 22), melanoma (n = 10), PDAC (n = 10), and HDs (n = 13), gated by excluding naïve T cells (CCR7^+^CD45RA^+^), show higher expression of the PD1^+^CD28^−^ phenotype in melanoma patients over HD. NSCLC patients show a higher frequency of less differentiated PD1^-^CD28^+^ and a lower incidence of more differentiated PD1^−^CD28^-^ among memory T cells either over HDs or the other tumors analyzed.* P* values were calculated by the Mann–Whitney unpaired two-sample test. *P ≤ 0.05,**P ≤ 0.01,***P ≤ 0.001,****P ≤ 0.0001. NS, not significant. B Percentage of CM, N, EM, and EMRA, as evaluated by CCR7 and CD45RA expression by flow cytometry, within the PD1/CD28 T-cell subsets. Analysis was performed in unstimulated ex vivo PBMC from NSCLC patients *(*n = 21, upper panels) and following short (5 h) (n = 14, meddle panels) or durable (48 h) (n = 7, lower panels) activation with anti-CD3 mAb. 

**Additional file 9:** **Figure S4.** In the tumor, the increase of PD-1^high^ impairs IFN-γ production only in the absence of CD28. A Proliferation ability, as evaluated by the fold increase proportion of PD1^+^CD28^−^ and PD1^+^CD28^+^ T cells after anti-CD3 mAb stimulation (48 h). B Representative dot-plot of PD1^high^ and PD1^low^ T-cell gating strategy. C Frequency (top panels) and functionality (upper panels) in PD1^high^ or PD1^low^ T cells, lacking or expressing CD28, from peripheral blood to the tumor site of NSCLC patients (n = 18). Intra-cellular GrzB, IFN-γ, and TNF-α expression were measured following anti-CD3 mAb activation (5-6 h) in the presence of protein transport inhibitors. *P* values were calculated by Wilcoxon rank test, with Bonferroni correction for multiple comparisons. * *P* ≤ 0.05,**P ≤ 0.01,***P ≤ 0.001. NS, not significant. NT, adjacent non-tumor tissue; T, tumor tissue. Graphs show median values with interquartile range.

**Additional file 10:** **Figure S5.** Although strongly reduced, expression of CD11a confers functional advantage to PD1^+^CD28^−^ over PD1^+^CD28^+^ T cells either in peripheral blood, NT, and tumor site. A-C Polyfunctional comparison between PD1^+^CD28^−^ and PD1^+^CD28^+^ T-cell subsets, evaluated within PBMC, NT or tumor site, in gated CD11a^high^ or CD11a^low/−^ cells (A, n = 10), ICOS^+^ or ICOS^−^ cells (B, n = 6) and CD137^+^ or CD137^−^ cells (C, n = 10). Simultaneous intra-cellular GrzB, IFN-γ, and TNF-α expression was measured following anti-CD3 mAb activation (5-6 h) in the presence of protein transport inhibitors. *P* values were calculated using the Wilcoxon rank test. * *P* ≤ 0.05, **P ≤ 0.01. NS, not significant. NT, adjacent non-tumor tissue; T, tumor tissue.

**Additional file 11:** **Figure S6.** PD1^+^CD28^−^ T-cell subset from PBMC of HDs is enriched in Ag-specific CMV^+^ CD8^+^ T cells that are functional when CD11a^+^ and CD137^+^. A Left, representative staining from one HD showing the proportion of unstimulated ex vivo PD1/CD28 subsets within total CD8^+^ T cells or within the CD8^+^CMV^+^ T cells. The percentage of positive expression is shown. Right, pooled results from 6 HDs. B Left, representative staining from one HD showing the quantification of unstimulated ex vivo CD8^+^ HLA-A2/CMV-dextramer^+^ T cells within the four PD1/CD28 subpopulations. Right, pooled results from 6 HDs. C Expression of CD11a^high^ and CD137^+^ T cells within different unstimulated ex vivo T-cell subsets, as indicated (n = 6). D Percentage of polyfunctional T cells, as evaluated by simultaneous intracellular production of GrzB, IFN-γ and TNF-α, within the T-cell subsets, following anti-CD3 mAb activation (5-6 h) in the presence of protein transport inhibitors, as indicated (n = 5). *P* values were calculated using Wilcoxon rank. * *P* ≤ 0.05. NS, not significant. Plots show median with interquartile range. 

**Additional file 12:** **Figure S7.** PD1, TIGIT and CTLA-4 inhibitory receptor expression increases from PBMC to tumor site. A Analysis of single PD1, TIGIT, TIM-3, CTLA-4 and LAG-3 expression in total CD8^+^ T cells, from matched unstimulated ex vivo PBMC, NT and tumor site, in NSCLC patients (n = 19). *P* values were calculated using Wilcoxon rank test, with Bonferroni correction for multiple comparisons. B Quantification of 32 possible combinations of the five IRs co-expression, in gated CD8^+^ T cells (n = 10). P values were calculated using the Friedman test between the three districts. *P ≤ 0.05,**P ≤ 0.01,***P ≤ 0.001. NS, not significant. C Correlation between the proportion of CD8^+^ T cells expressing each IR, in PBMC *vs* NT (upper panels) and in PBMC *vs* tumor tissue (lower panels). Pearson correlation was used to compare variables. P, PBMC; N and NT, adjacent non-tumor tissue; T, tumor tissue. Plots show median values with interquartile range.

**Additional file 13:** **Figure S8.** IR expression and polyfunctionality within PD1^+^CD28^−^ or PD1^+^CD28^+^ subsets, in cells from PBMC, NT, and tumor site. A Comparison of single TIGIT, CTLA-4, TIM-3, and LAG-3 IR expression between PD1^+^CD28^−^ and PD1^+^CD28^+^ T-cell subsets, in unstimulated ex vivo cells from PBMC, NT and tumor site, in 19 NSCLC patients. P values were calculated using the Wilcoxon rank test. B Polyfunctionality of the 16 IR subgroups within PD1^+^CD28^−^ or PD1^+^CD28^+^ subsets, in cells from PBMC, NT, and tumor site from 10 NSCLC patients. C Heat dot plot illustrating the combination of frequency (dot dimension) and polyfunctionality (dot plot color) within 16 distinct immune receptor (IR) subgroups, each representing a unique combination, within PD1^+^CD28^−^ and PD1^+^CD28^+^ subsets. Data was obtained from PBMC, NT and tumor site of 10 NSCLC patients. Functionality was summarized using the median values for each district. Comparative analysis was performed between IR1 (quadruple-negative) and other subgroups (IR2-IR16) in PBMC and tumor site. Orange dots symbolize subgroups with lower functionality relative to IR1, while green dots indicate higher functionality compared to the IR1 subgroup. Small grey dots show the absence while large black dots indicate the presence of the corresponding inhibitory receptors. P values were calculated using the Friedman test between the three districts. * *P* ≤ 0.05, **P ≤ 0.01,***P ≤ 0.001. NS, not significant. P, PBMC; N and NT, adjacent non-tumor tissue; T, tumor tissue. Graphs shown median with interquartile range.

**Additional file 14:** **Figure S9.** PD1^+^CD28^−^ and PD1^+^CD28^+^ T-cell subsets are mutual players of the CXCL13/CXCR5 axis. A Intracellular CXCL13 expression, evaluated by flow cytometry, in different T-cell subsets, as indicated, unstimulated ex vivo (upper panels, NT, *n* = 5; T, *n* = 12) or following 6-days in-vitro expansion with anti-CD3 mAb plus TGFβ in the presence of protein transport inhibitors (lower panels, NT, *n* = 6; T, n = 7), in NSCLC patients (Wilcoxon rank test, with Bonferroni correction). B Intracellular CXCL13 expression in different T-cell subsets, as indicated, following 6-days in-vitro expansion with anti-CD3 mAb plus TGFβ (NT, n = 6; T, n = 7) (Wilcoxon rank test, with Bonferroni correction). C Representative flow cytometry gating strategy showing intracellular CXCL13 expression in different T-cell populations, following 6-days anti-CD3 mAb plus TGFβ stimulation. Percentage of positive expression is shown. D Expression of CXCR5 in different T-cell subsets, as indicated, from unstimulated ex vivo PBMC (*n* = 12), NT (*n* = 5) and tumor site (*n* = 12) of NSCLC patients (Wilcoxon rank test, with Bonferroni correction). * *P* ≤ 0.05, **P ≤ 0.01, NS, not significant. NT, adjacent non-tumor tissue; T, tumor tissue.

**Additional file 15:** **Figure S10.** Expression of selected T-cell immune markers in the different CD8^+^ PD1^+^CD28^−^ and PD1^+^CD28^+^ T-cell subsets. A UMAP showing the expression of selected mAbs from the implemented Immune Discovery Panel, in gated total live cells. B Violin-plot showing the expression level of selected mAbs in the clusters identified within PD1^+^CD28^−^ and PD1^+^CD28^+^ T-cell subsets. C Heatmap-dot plot showing the percentage and the expression level of selected genes.

## Data Availability

Datasets supporting the conclusion of this article are available in Zenodo (https://zenodo.org/) under the DOIs "10.5281/zenodo.8186193" (RNA-Seq raw data from Ag-specific T-cell clones part 1), "10.5281/zenodo.8186516" (RNA-Seq raw data from Ag-specific T-cell clones part 2) and "10.5281/zenodo.8186224" (scRNA-Seq raw data of lymphocytes from the three districts scRNA-Seq), and are freely available upon request. Research data are stored in an Institutional repository and will be shared upon request to the Corresponding Author.

## Abbreviations

Ag: Antigen
CM: Central memory
CR: Complete Response
CTLA-4: Cytotoxic T lymphocyte antigen 4
DEG: Differential expression genes
EMRA: Terminal-differentiated effector-memory
EM: Effector-memory
E:T: Effector:Target
GrzB: Granzyme B
HDs: Healthy donors
HS: Human serum
ICB: Immune-checkpoint blockade
IFN: Interferon
IRs: Inhibitory receptors
LAG-3: Lymphocyte activation gene 3
LUAD: Lung adenocarcinoma
N: Naïve
NKT: Innate-like T invariant
NR: Non-Responder
NSCLC: Non-small cell lung-cancer
NT: Adjacent non-tumor tissue
mAb: Monoclonal antibody
OS: Overall survival
P: Peripheral blood
PBMC: Peripheral blood mononuclear cell
PD-1: Programmed cell death protein 1
PD-L1: Programmed death-ligand 1
PD: Progressive Disease
PDAC: Pancreas ductal adenocarcinoma cancer
PR: Partial Response
QN: Quadruple negative
QP: Quadruple positive
R: Responder
rIL-2: Recombinant Interleukin-2
RNA-Seq: RNA-based sequencing
scRNA-Seq: Single cell RNA-based sequencing
T: Tumor site
TCGA: The cancer genome atlas
TNF: Tumor necrosis factor
TIGIT: T-cell immunoreceptor with Ig and ITIM domains
TIM-3: T-cell immunoglobulin domain and mucin domain-3
T_RM_: Tissue-resident memory T cells
Vs: Versus
