# Supplementary material for: CD28/PD1 co-expression: dual impact on CD8+ T cells in peripheral blood and tumor tissue, and its significance in NSCLC patients' survival and ICB response
Source: J Exp Clin Cancer Res. 2023 Oct 28;42:287. doi: 10.1186/s13046-023-02846-3 (PMC10612243; doi:10.1186/s13046-023-02846-3)

Figure S1. Gene Set Enrichment Analysis (GSEA) of Exhaustion and Pre-exhaustion Gene Signatures in CD28- *vs* CD28+ T-cell clones

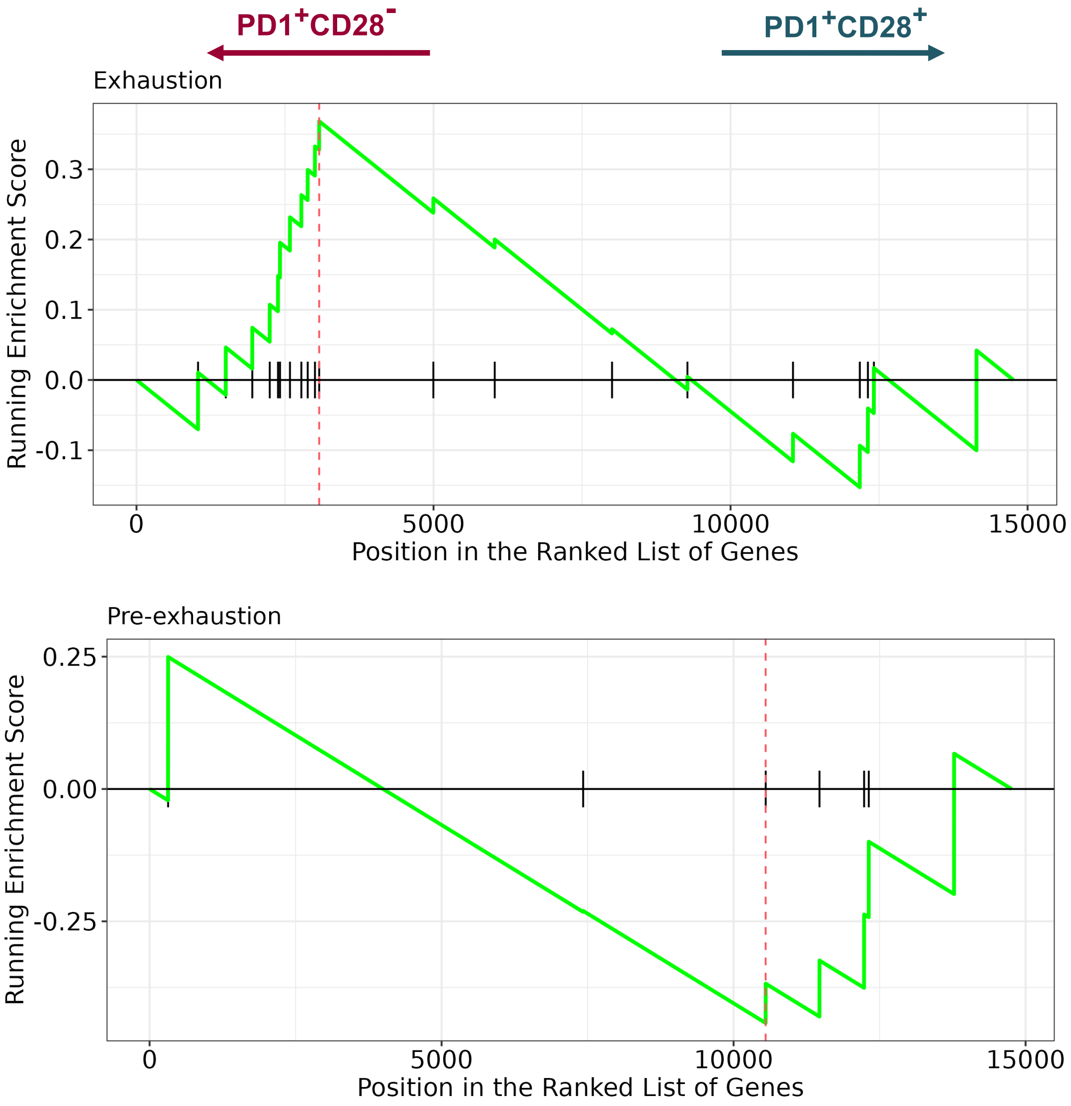

Supplement: Supplementary file 6 — Additional file 6: Figure S1. Gene Set Enrichment Analysis (GSEA) of Exhaustion and Pre-exhaustion Gene Signatures in CD28- vs CD28+ T-cell clones. GSEA depicting the distribution of Exhaustion and Pre-exhaustion gene signatures (Chu Y et al., Nat Med. doi: 10.1038/s41591-023-02371-y), within the differentially expressed gene list resulting from the CD28− vs CD28+ clones’ comparison. Genes are ranked based on the DESeq2 Wald test statistic (“stat”). The plots display the running sum of the Enrichment Score (ES) across the gene list (green line) and ES peak (red dashed line). GSEA reveals a clear trend for a higher expression of an “exhausted” transcriptional signature, while “pre-exhaustion” genes were more correlated with CD28+ cells. [file 13046_2023_2846_MOESM6_ESM.pdf]
