# Supplementary material for: CD28/PD1 co-expression: dual impact on CD8+ T cells in peripheral blood and tumor tissue, and its significance in NSCLC patients' survival and ICB response
Source: J Exp Clin Cancer Res. 2023 Oct 28;42:287. doi: 10.1186/s13046-023-02846-3 (PMC10612243; doi:10.1186/s13046-023-02846-3)

Palermo et al. Figure S2. Gating strategy of representative immunophenotyping panels for the staining with mAbs employed in the study

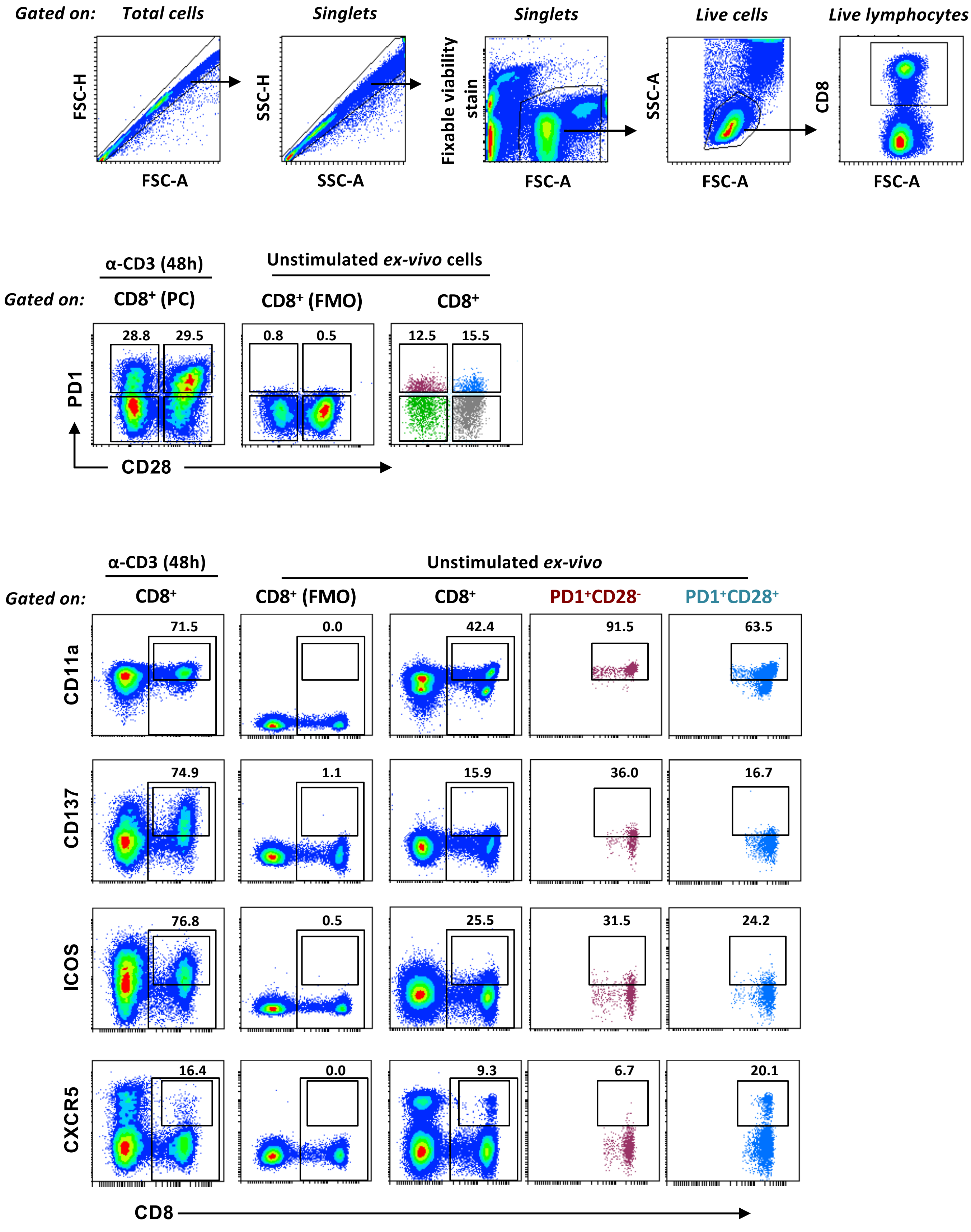

continued

Palermo et al. Figure S2 (*continued*)

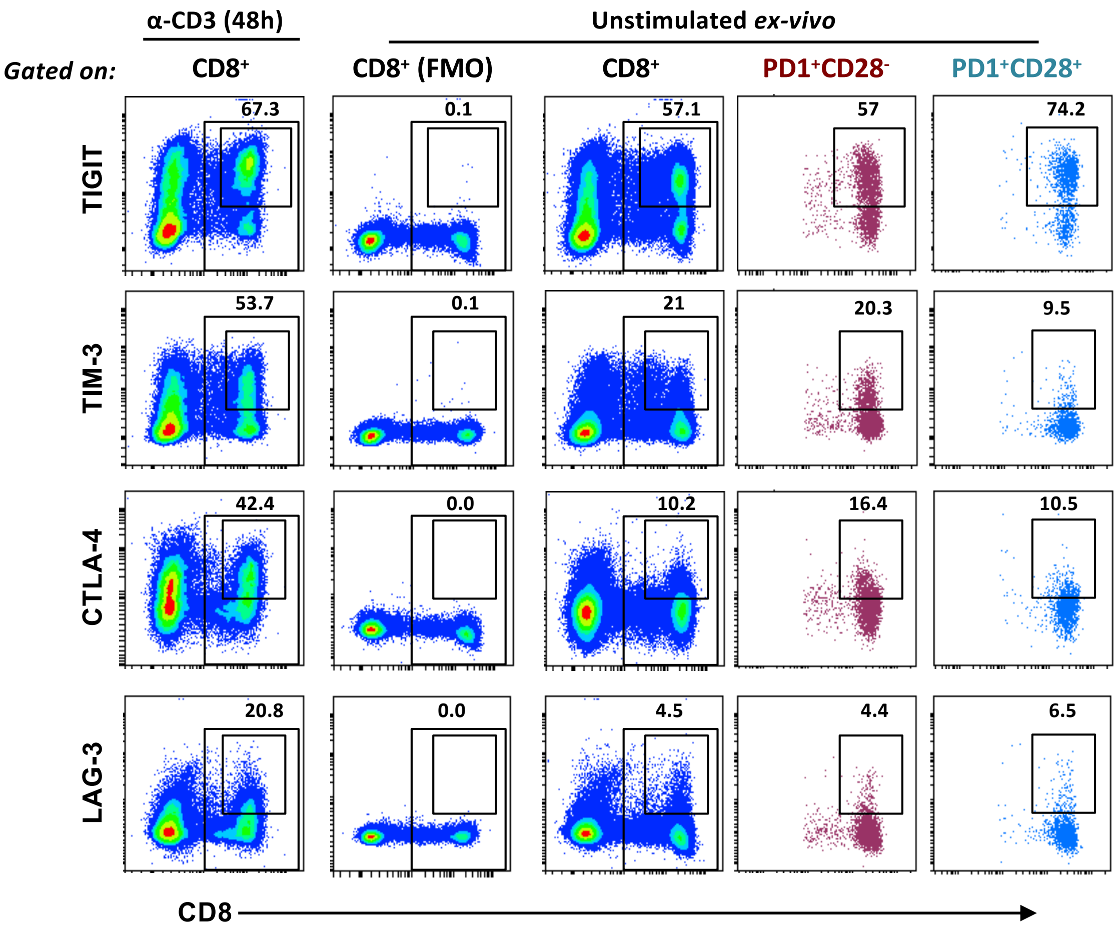

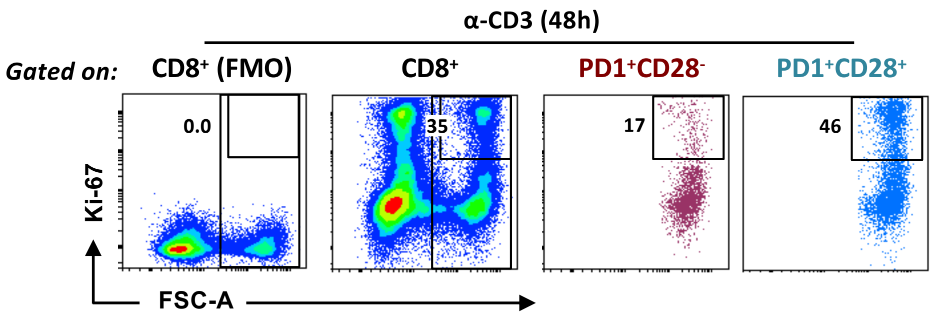

Supplement: Supplementary file 7 — Additional file 7: Figure S2. Gating strategy of representative immunophenotyping panels for the staining with mAbs employed in the study. Representative dot-plots from NSCLC patients, as evaluated by multicolor flow cytometry gated on total CD8+ T cells and PD1/CD28 subsets, either unstimulated or stimulated with anti-CD3 mAb. FMO, fluorescence minus one control. [file 13046_2023_2846_MOESM7_ESM.pdf]
