# Supplementary material for: CD28/PD1 co-expression: dual impact on CD8+ T cells in peripheral blood and tumor tissue, and its significance in NSCLC patients' survival and ICB response
Source: J Exp Clin Cancer Res. 2023 Oct 28;42:287. doi: 10.1186/s13046-023-02846-3 (PMC10612243; doi:10.1186/s13046-023-02846-3)

Figure S3. The T-cell differentiation stage impacts the circulating CD28/PD1 subset distribution and changes as a function of the activation interval

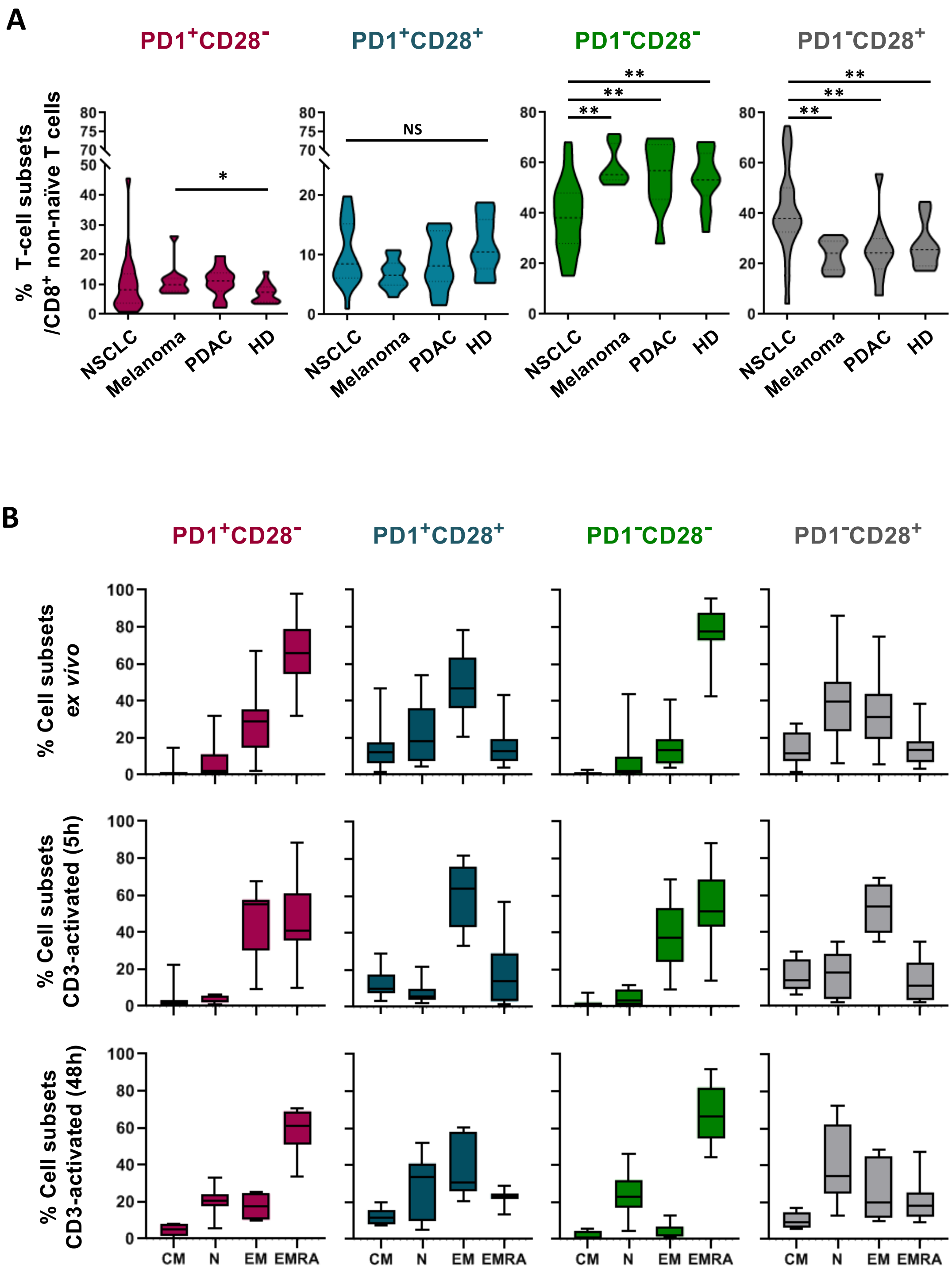

Supplement: Supplementary file 8 — Additional file 8: Figure S3. The T-cell differentiation stage impacts the circulating CD28/PD-1 subset distribution and changes as a function of the activation interval. A Unstimulated ex vivo PBMC from NSCLC (n = 22), melanoma (n = 10), PDAC (n = 10), and HDs (n = 13), gated by excluding naïve T cells (CCR7+CD45RA+), show higher expression of the PD1+CD28− phenotype in melanoma patients over HD. NSCLC patients show a higher frequency of less differentiated PD1-CD28+ and a lower incidence of more differentiated PD1−CD28- among memory T cells either over HDs or the other tumors analyzed. P values were calculated by the Mann–Whitney unpaired two-sample test. *P ≤ 0.05,**P ≤ 0.01,***P ≤ 0.001,****P ≤ 0.0001. NS, not significant. B Percentage of CM, N, EM, and EMRA, as evaluated by CCR7 and CD45RA expression by flow cytometry, within the PD1/CD28 T-cell subsets. Analysis was performed in unstimulated ex vivo PBMC from NSCLC patients (n = 21, upper panels) and following short (5 h) (n = 14, meddle panels) or durable (48 h) (n = 7, lower panels) activation with anti-CD3 mAb. [file 13046_2023_2846_MOESM8_ESM.pdf]
