# Supplementary material for: CD28/PD1 co-expression: dual impact on CD8+ T cells in peripheral blood and tumor tissue, and its significance in NSCLC patients' survival and ICB response
Source: J Exp Clin Cancer Res. 2023 Oct 28;42:287. doi: 10.1186/s13046-023-02846-3 (PMC10612243; doi:10.1186/s13046-023-02846-3)

Figure S4. In the tumor, the increase of PD-1^{high} impairs IFN-ɣ production only in the absence of CD28.

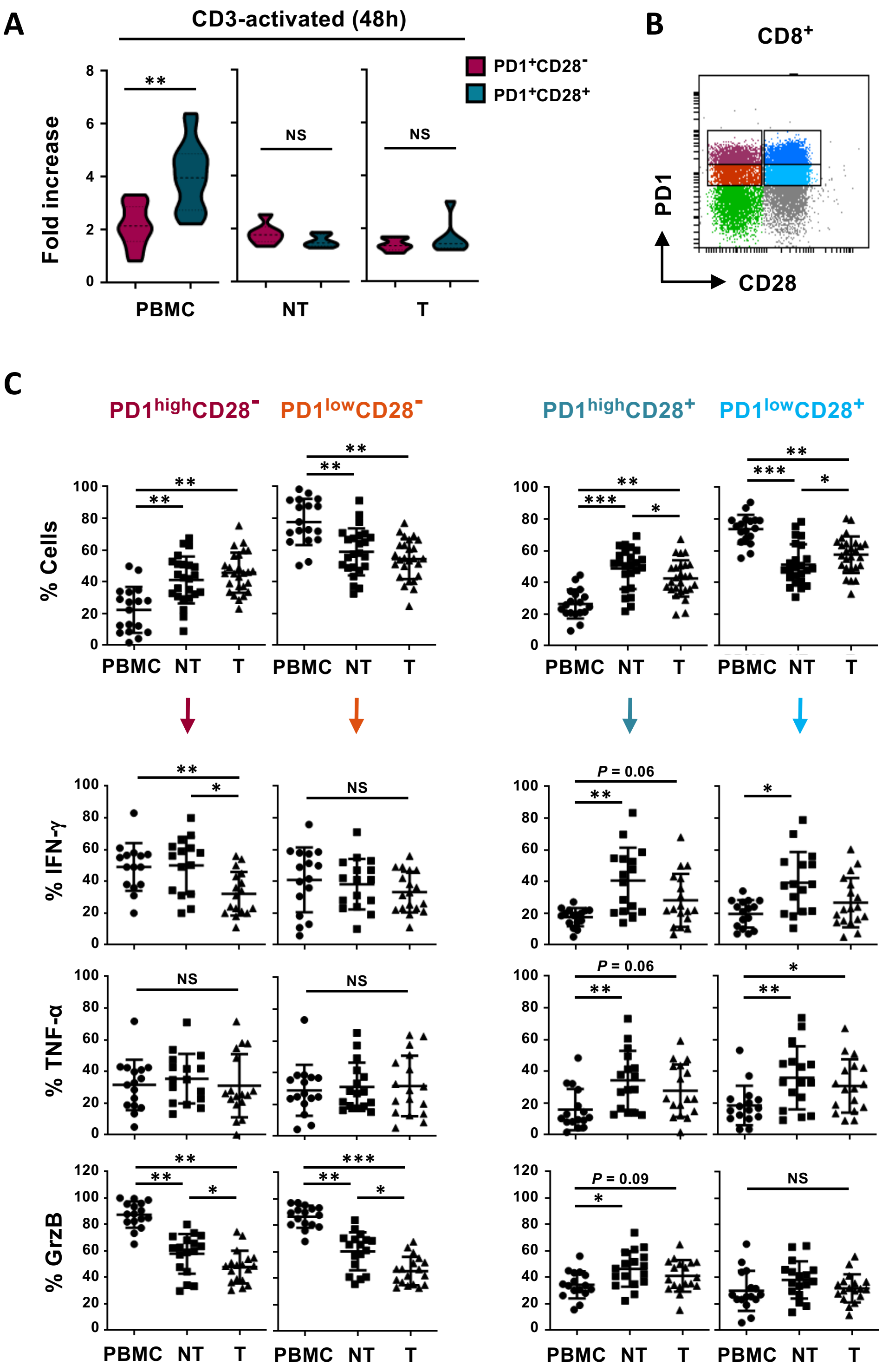

Supplement: Supplementary file 9 — Additional file 9: Figure S4. In the tumor, the increase of PD-1high impairs IFN-γ production only in the absence of CD28. A Proliferation ability, as evaluated by the fold increase proportion of PD1+CD28− and PD1+CD28+ T cells after anti-CD3 mAb stimulation (48 h). B Representative dot-plot of PD1high and PD1low T-cell gating strategy. C Frequency (top panels) and functionality (upper panels) in PD1high or PD1low T cells, lacking or expressing CD28, from peripheral blood to the tumor site of NSCLC patients (n = 18). Intra-cellular GrzB, IFN-γ, and TNF-α expression were measured following anti-CD3 mAb activation (5-6 h) in the presence of protein transport inhibitors. P values were calculated by Wilcoxon rank test, with Bonferroni correction for multiple comparisons. * P ≤ 0.05,**P ≤ 0.01,***P ≤ 0.001. NS, not significant. NT, adjacent non-tumor tissue; T, tumor tissue. Graphs show median values with interquartile range. [file 13046_2023_2846_MOESM9_ESM.pdf]
