# Supplementary material for: CD28/PD1 co-expression: dual impact on CD8+ T cells in peripheral blood and tumor tissue, and its significance in NSCLC patients' survival and ICB response
Source: J Exp Clin Cancer Res. 2023 Oct 28;42:287. doi: 10.1186/s13046-023-02846-3 (PMC10612243; doi:10.1186/s13046-023-02846-3)

Figure S5. Although strongly reduced, expression of CD11a confers functional advantage to PD1⁺CD28⁻ over PD1⁺CD28⁺ T cells either in peripheral blood, NT and tumor site.

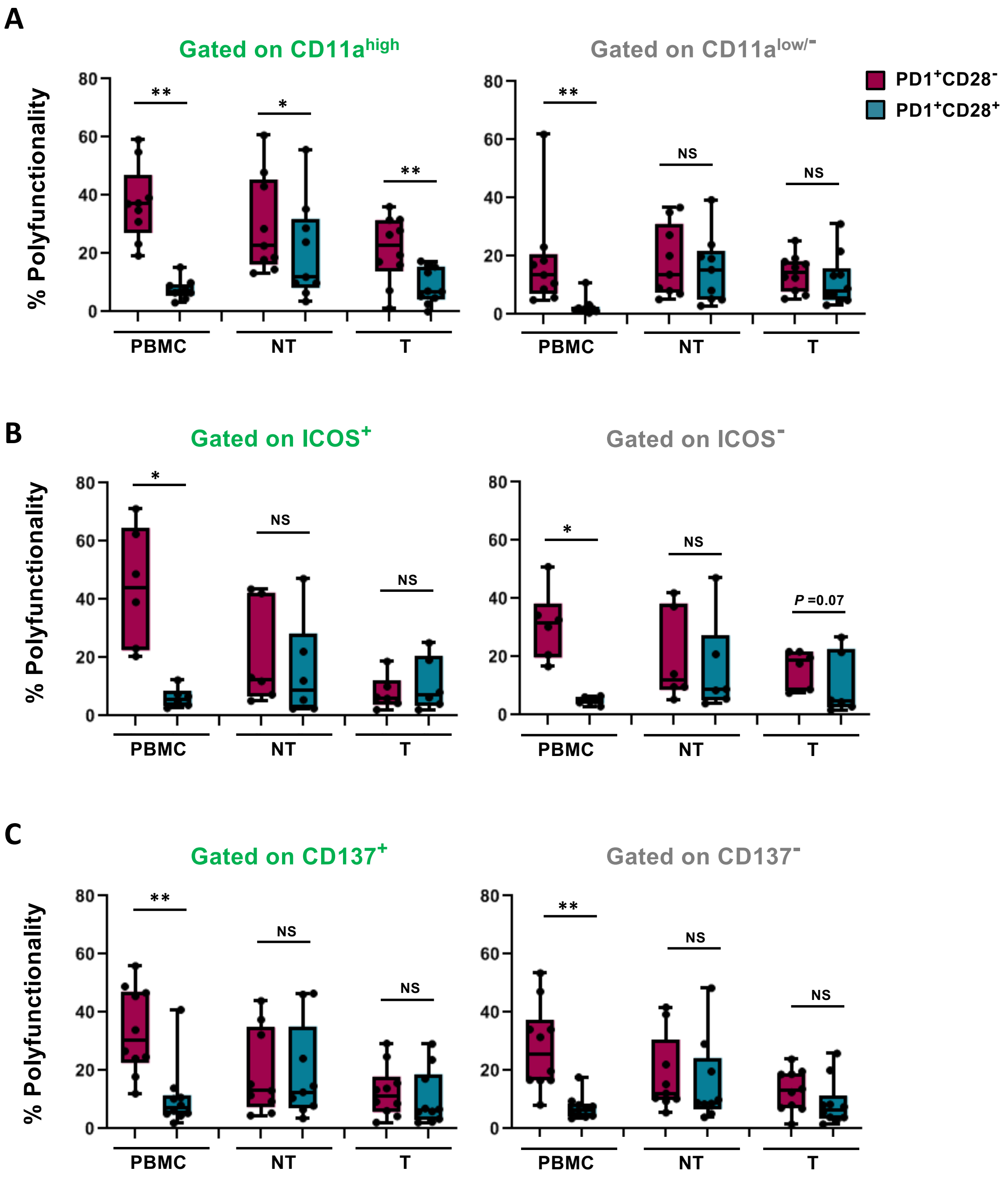

Supplement: Supplementary file 10 — Additional file 10: Figure S5. Although strongly reduced, expression of CD11a confers functional advantage to PD1+CD28− over PD1+CD28+ T cells either in peripheral blood, NT, and tumor site. A-C Polyfunctional comparison between PD1+CD28− and PD1+CD28+ T-cell subsets, evaluated within PBMC, NT or tumor site, in gated CD11ahigh or CD11alow/− cells (A, n = 10), ICOS+ or ICOS− cells (B, n = 6) and CD137+ or CD137− cells (C, n = 10). Simultaneous intra-cellular GrzB, IFN-γ, and TNF-α expression was measured following anti-CD3 mAb activation (5-6 h) in the presence of protein transport inhibitors. P values were calculated using the Wilcoxon rank test. * P ≤ 0.05, **P ≤ 0.01. NS, not significant. NT, adjacent non-tumor tissue; T, tumor tissue. [file 13046_2023_2846_MOESM10_ESM.pdf]
