# Supplementary material for: CD28/PD1 co-expression: dual impact on CD8+ T cells in peripheral blood and tumor tissue, and its significance in NSCLC patients' survival and ICB response
Source: J Exp Clin Cancer Res. 2023 Oct 28;42:287. doi: 10.1186/s13046-023-02846-3 (PMC10612243; doi:10.1186/s13046-023-02846-3)

Figure S6. PD1⁺CD28⁻ T-cell subset from PBMC of HDs is enriched in Ag-specific CMV⁺ CD8⁺ T cells that are functional when CD11a⁺ and CD137⁺.

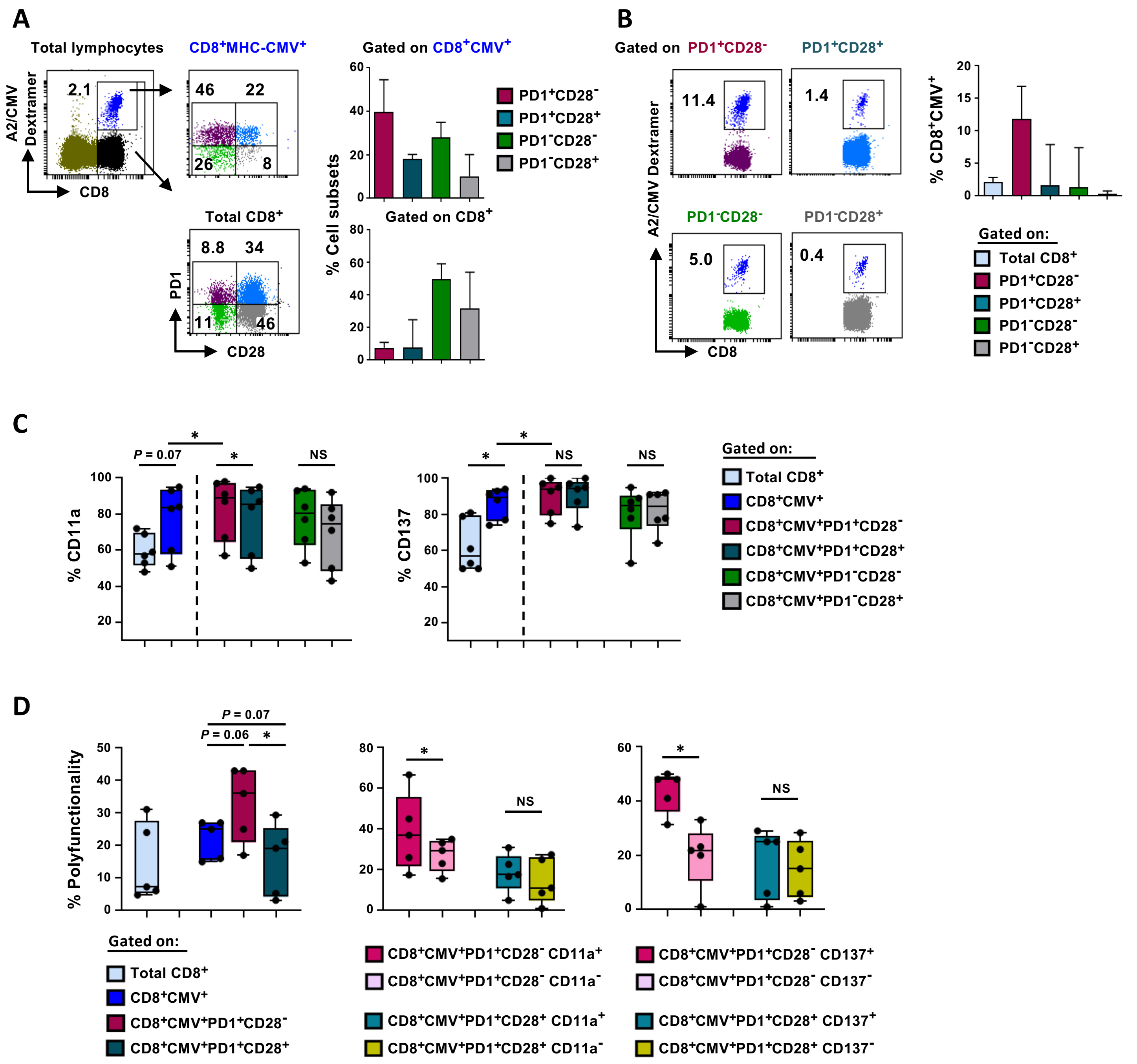

Supplement: Supplementary file 11 — Additional file 11: Figure S6. PD1+CD28− T-cell subset from PBMC of HDs is enriched in Ag-specific CMV+ CD8+ T cells that are functional when CD11a+ and CD137+. A Left, representative staining from one HD showing the proportion of unstimulated ex vivo PD1/CD28 subsets within total CD8+ T cells or within the CD8+CMV+ T cells. The percentage of positive expression is shown. Right, pooled results from 6 HDs. B Left, representative staining from one HD showing the quantification of unstimulated ex vivo CD8+ HLA-A2/CMV-dextramer+ T cells within the four PD1/CD28 subpopulations. Right, pooled results from 6 HDs. C Expression of CD11ahigh and CD137+ T cells within different unstimulated ex vivo T-cell subsets, as indicated (n = 6). D Percentage of polyfunctional T cells, as evaluated by simultaneous intracellular production of GrzB, IFN-γ and TNF-α, within the T-cell subsets, following anti-CD3 mAb activation (5-6 h) in the presence of protein transport inhibitors, as indicated (n = 5). P values were calculated using Wilcoxon rank. * P ≤ 0.05. NS, not significant. Plots show median with interquartile range. [file 13046_2023_2846_MOESM11_ESM.pdf]
