# Supplementary figures and images for: CD28/PD1 co-expression: dual impact on CD8+ T cells in peripheral blood and tumor tissue, and its significance in NSCLC patients' survival and ICB response
Source: J Exp Clin Cancer Res. 2023 Oct 28;42:287. doi: 10.1186/s13046-023-02846-3 (PMC10612243; doi:10.1186/s13046-023-02846-3)

Figure S7. PD1, TIGIT and CTLA-4 inhibitory receptor expression increases from PBMC to tumor site.

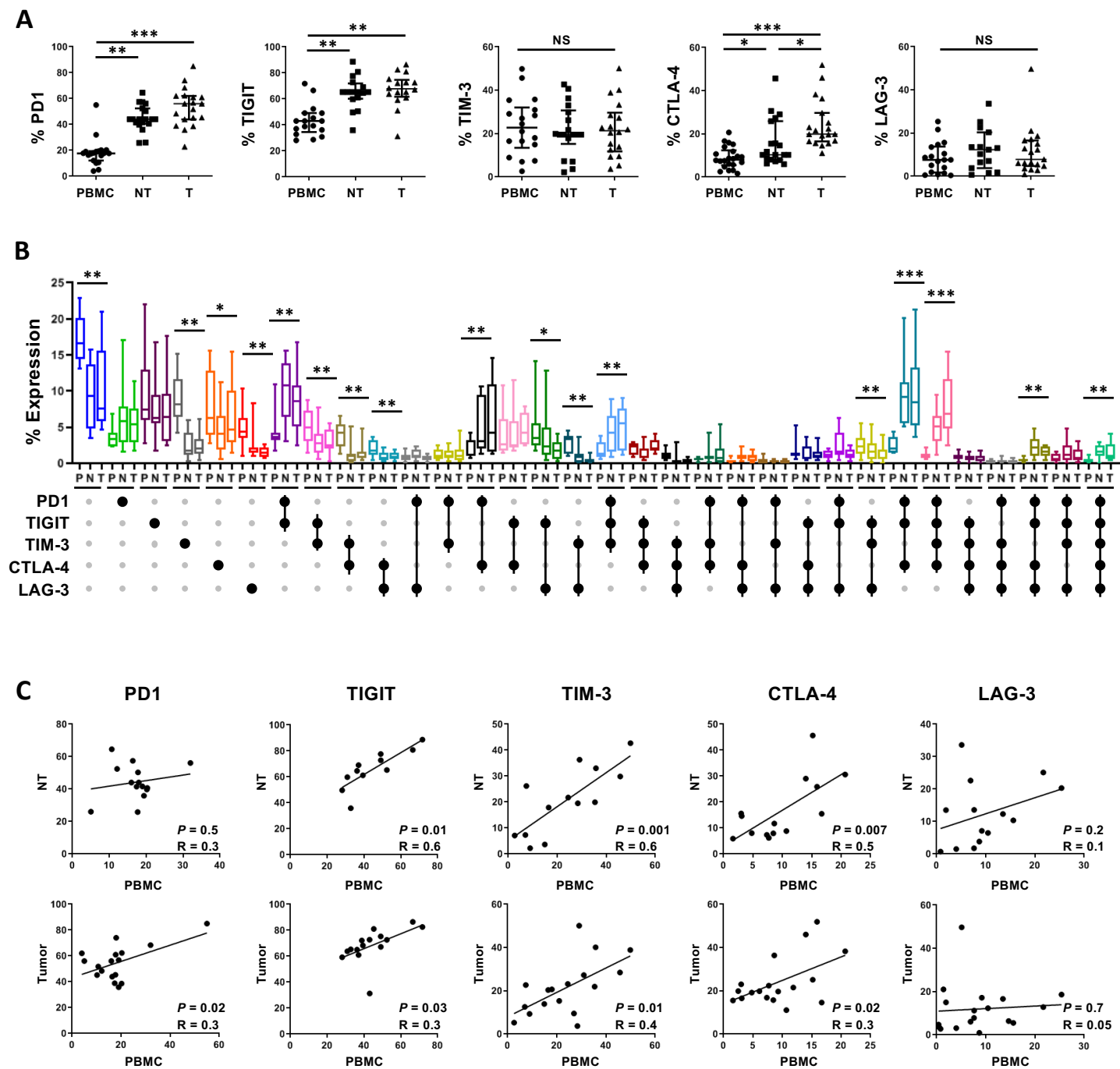

Supplement: Supplementary file 12 — Additional file 12: Figure S7. PD1, TIGIT and CTLA-4 inhibitory receptor expression increases from PBMC to tumor site. A Analysis of single PD1, TIGIT, TIM-3, CTLA-4 and LAG-3 expression in total CD8+ T cells, from matched unstimulated ex vivo PBMC, NT and tumor site, in NSCLC patients (n = 19). P values were calculated using Wilcoxon rank test, with Bonferroni correction for multiple comparisons. B Quantification of 32 possible combinations of the five IRs co-expression, in gated CD8+ T cells (n = 10). P values were calculated using the Friedman test between the three districts. *P ≤ 0.05,**P ≤ 0.01,***P ≤ 0.001. NS, not significant. C Correlation between the proportion of CD8+ T cells expressing each IR, in PBMC vs NT (upper panels) and in PBMC vs tumor tissue (lower panels). Pearson correlation was used to compare variables. P, PBMC; N and NT, adjacent non-tumor tissue; T, tumor tissue. Plots show median values with interquartile range. [file 13046_2023_2846_MOESM12_ESM.pdf]
