# Supplementary material for: CD28/PD1 co-expression: dual impact on CD8+ T cells in peripheral blood and tumor tissue, and its significance in NSCLC patients' survival and ICB response
Source: J Exp Clin Cancer Res. 2023 Oct 28;42:287. doi: 10.1186/s13046-023-02846-3 (PMC10612243; doi:10.1186/s13046-023-02846-3)

Figure S8. IRs expression and polyfunctionality within PD1+CD28- or PD1+CD28+ subsets, in cells from PBMC, NT and tumor site.

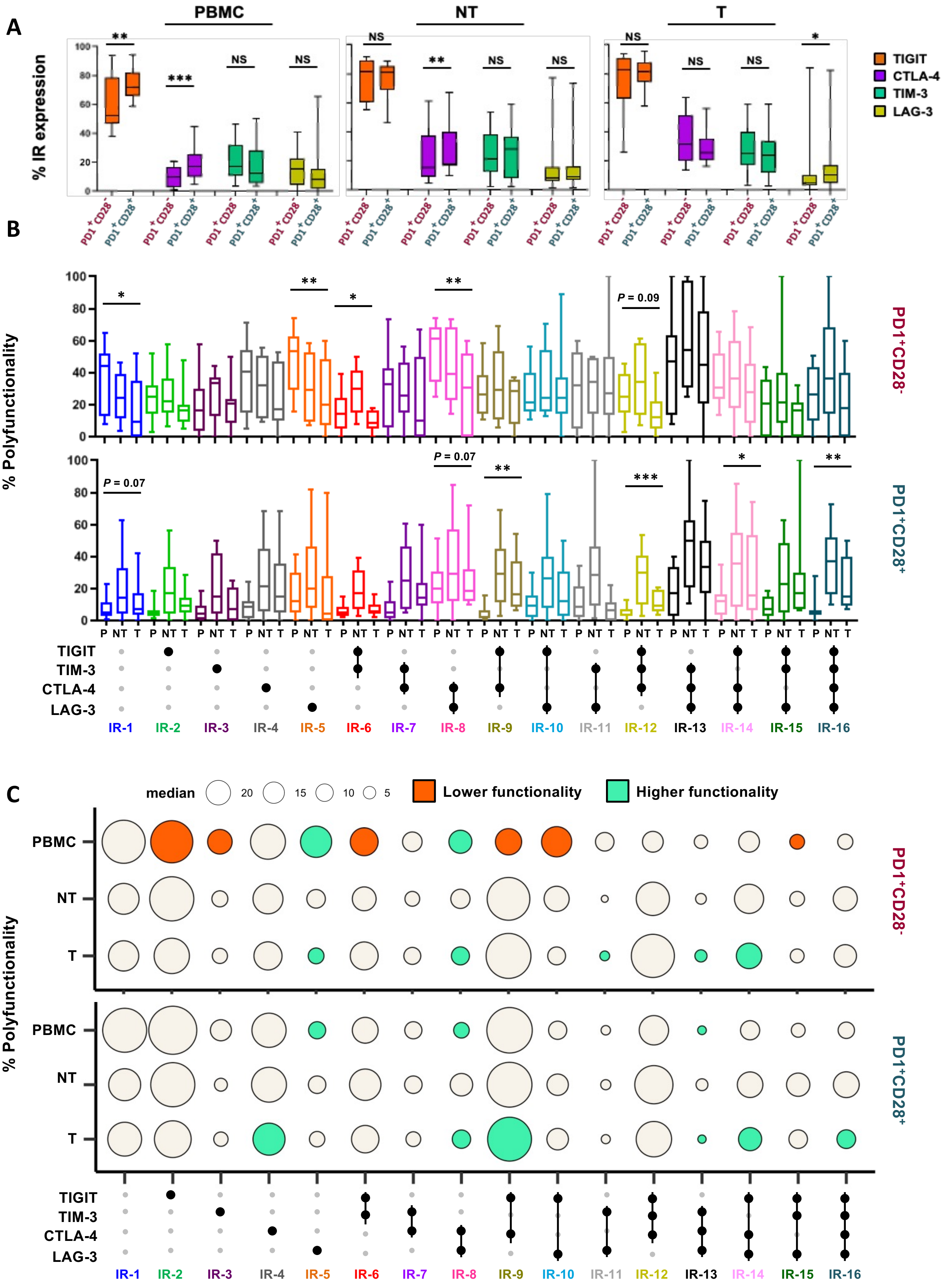

Supplement: Supplementary file 13 — Additional file 13: Figure S8. IR expression and polyfunctionality within PD1+CD28− or PD1+CD28+ subsets, in cells from PBMC, NT, and tumor site. A Comparison of single TIGIT, CTLA-4, TIM-3, and LAG-3 IR expression between PD1+CD28− and PD1+CD28+ T-cell subsets, in unstimulated ex vivo cells from PBMC, NT and tumor site, in 19 NSCLC patients. P values were calculated using the Wilcoxon rank test. B Polyfunctionality of the 16 IR subgroups within PD1+CD28− or PD1+CD28+ subsets, in cells from PBMC, NT, and tumor site from 10 NSCLC patients. C Heat dot plot illustrating the combination of frequency (dot dimension) and polyfunctionality (dot plot color) within 16 distinct immune receptor (IR) subgroups, each representing a unique combination, within PD1+CD28− and PD1+CD28+ subsets. Data was obtained from PBMC, NT and tumor site of 10 NSCLC patients. Functionality was summarized using the median values for each district. Comparative analysis was performed between IR1 (quadruple-negative) and other subgroups (IR2-IR16) in PBMC and tumor site. Orange dots symbolize subgroups with lower functionality relative to IR1, while green dots indicate higher functionality compared to the IR1 subgroup. Small grey dots show the absence while large black dots indicate the presence of the corresponding inhibitory receptors. P values were calculated using the Friedman test between the three districts. * P ≤ 0.05, **P ≤ 0.01,***P ≤ 0.001. NS, not significant. P, PBMC; N and NT, adjacent non-tumor tissue; T, tumor tissue. Graphs shown median with interquartile range. [file 13046_2023_2846_MOESM13_ESM.pdf]
