# Supplementary material for: CD28/PD1 co-expression: dual impact on CD8+ T cells in peripheral blood and tumor tissue, and its significance in NSCLC patients' survival and ICB response
Source: J Exp Clin Cancer Res. 2023 Oct 28;42:287. doi: 10.1186/s13046-023-02846-3 (PMC10612243; doi:10.1186/s13046-023-02846-3)

Figure S9. PD1$^+$CD28$^-$ and PD1$^+$CD28$^+$ T-cell subsets are mutual players of the CXCL13/CXCR5 axis.

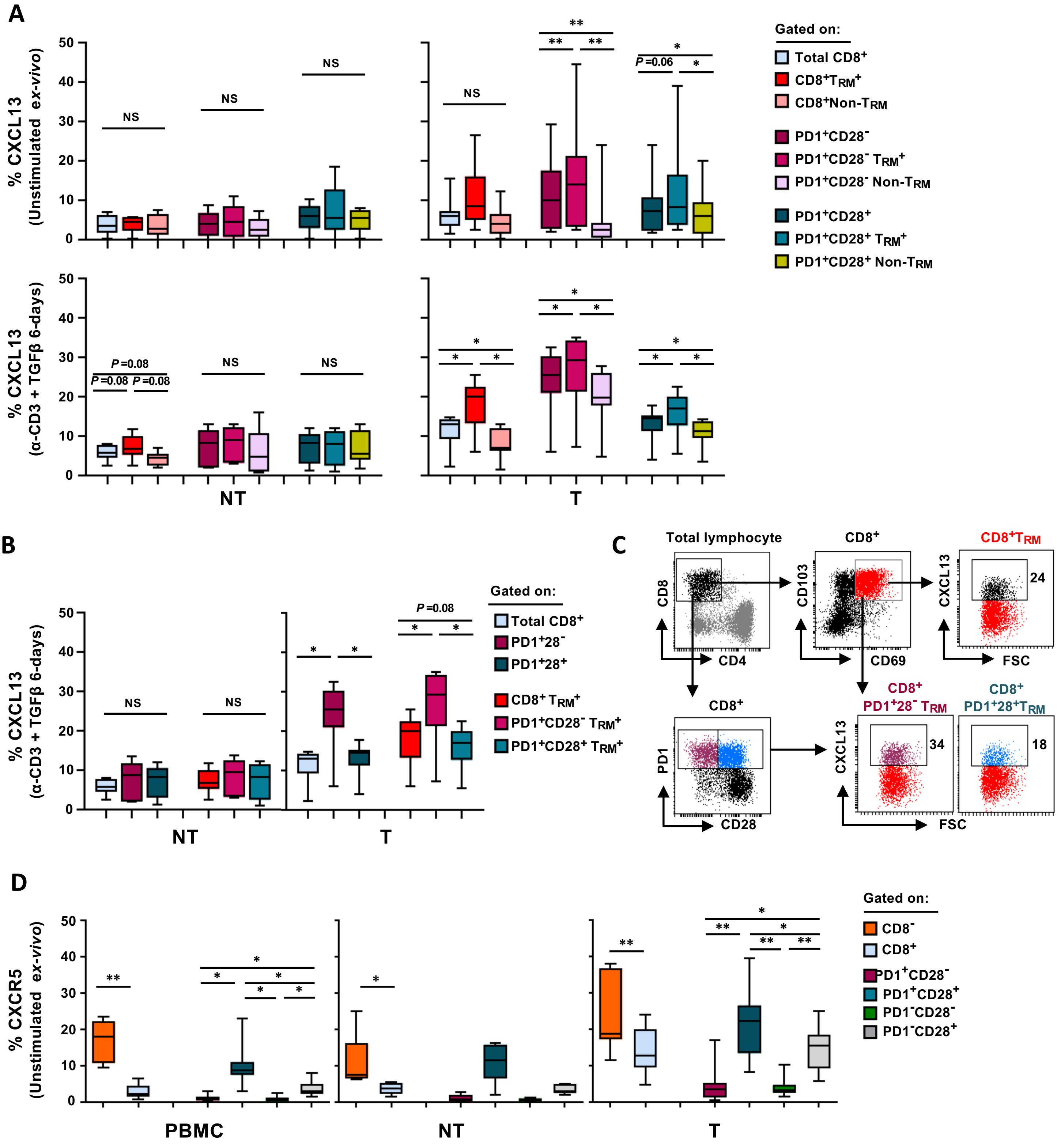

Supplement: Supplementary file 14 — Additional file 14: Figure S9. PD1+CD28− and PD1+CD28+ T-cell subsets are mutual players of the CXCL13/CXCR5 axis. A Intracellular CXCL13 expression, evaluated by flow cytometry, in different T-cell subsets, as indicated, unstimulated ex vivo (upper panels, NT, n = 5; T, n = 12) or following 6-days in-vitro expansion with anti-CD3 mAb plus TGFβ in the presence of protein transport inhibitors (lower panels, NT, n = 6; T, n = 7), in NSCLC patients (Wilcoxon rank test, with Bonferroni correction). B Intracellular CXCL13 expression in different T-cell subsets, as indicated, following 6-days in-vitro expansion with anti-CD3 mAb plus TGFβ (NT, n = 6; T, n = 7) (Wilcoxon rank test, with Bonferroni correction). C Representative flow cytometry gating strategy showing intracellular CXCL13 expression in different T-cell populations, following 6-days anti-CD3 mAb plus TGFβ stimulation. Percentage of positive expression is shown. D Expression of CXCR5 in different T-cell subsets, as indicated, from unstimulated ex vivo PBMC (n = 12), NT (n = 5) and tumor site (n = 12) of NSCLC patients (Wilcoxon rank test, with Bonferroni correction). * P ≤ 0.05, **P ≤ 0.01, NS, not significant. NT, adjacent non-tumor tissue; T, tumor tissue. [file 13046_2023_2846_MOESM14_ESM.pdf]
