# Supplementary material for: CD28/PD1 co-expression: dual impact on CD8+ T cells in peripheral blood and tumor tissue, and its significance in NSCLC patients' survival and ICB response
Source: J Exp Clin Cancer Res. 2023 Oct 28;42:287. doi: 10.1186/s13046-023-02846-3 (PMC10612243; doi:10.1186/s13046-023-02846-3)

Figure S10. Expression of selected T-cell immune markers in the different CD8$^+$ PD1$^+$CD28$^-$ and PD1$^+$CD28$^+$.

A

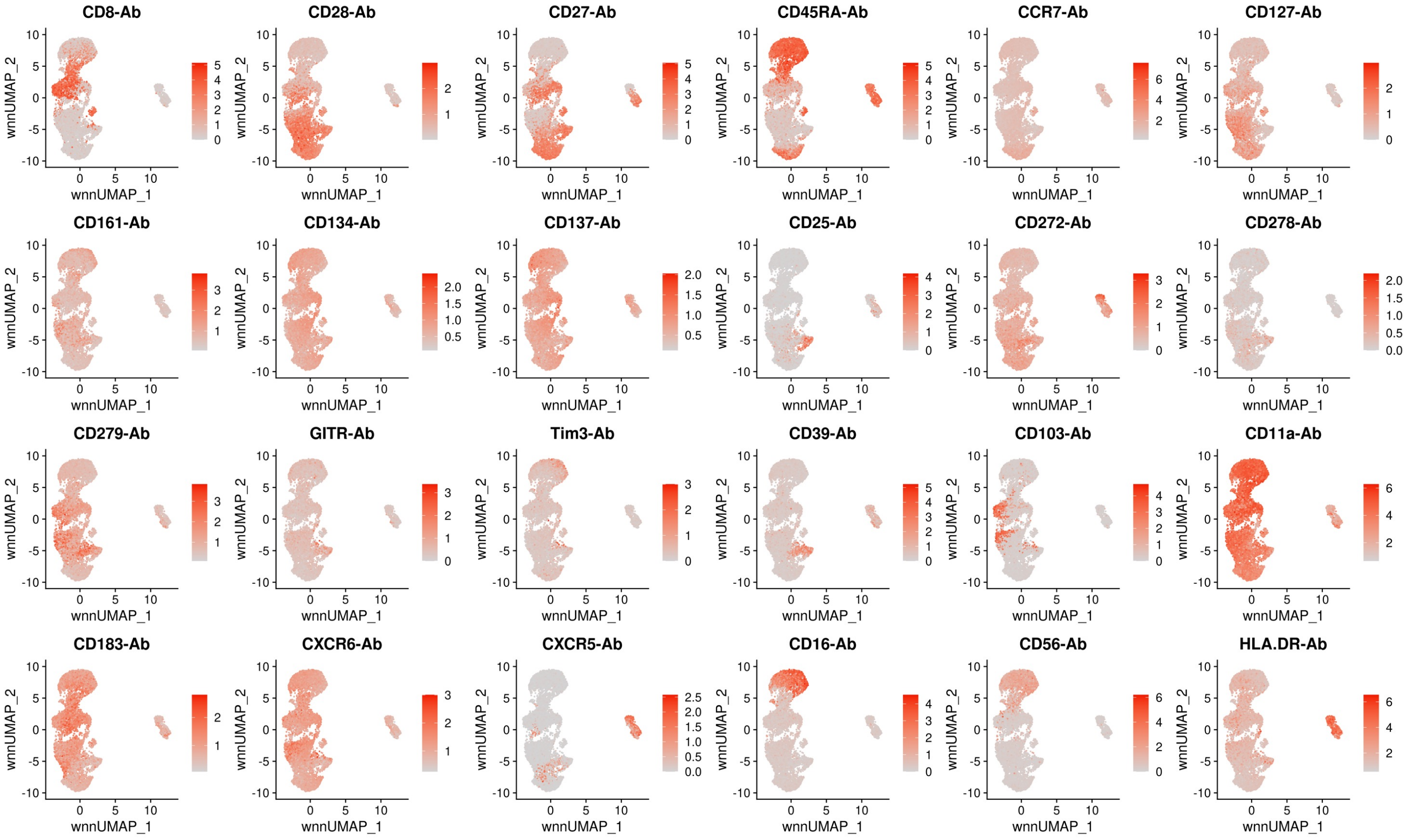

B

Gated on CD8$^+$ PD1$^+$CD28$^-$

Gated on CD8$^+$ PD1$^+$CD28$^+$

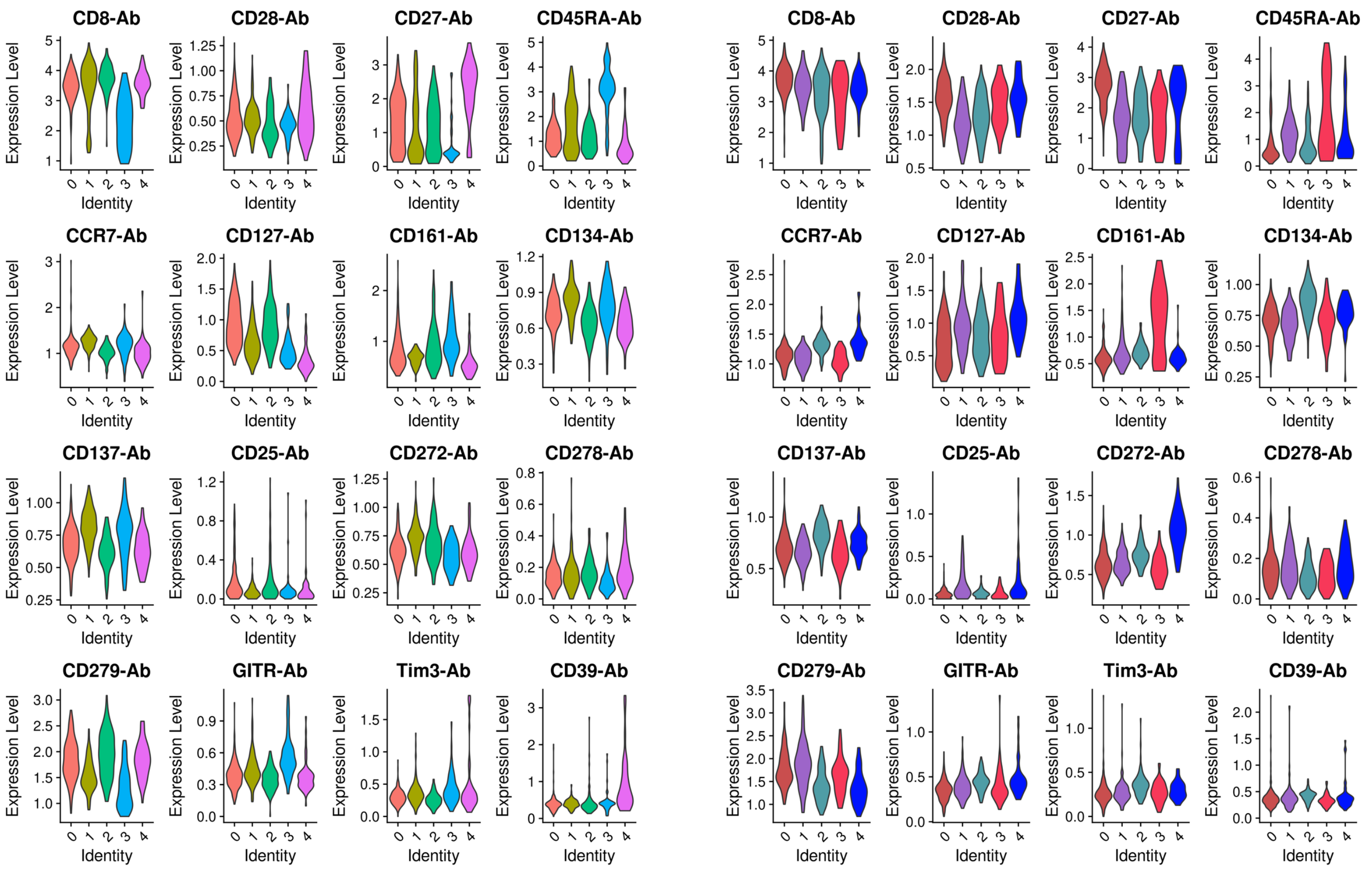

continued

Figure S10 (continued)

B

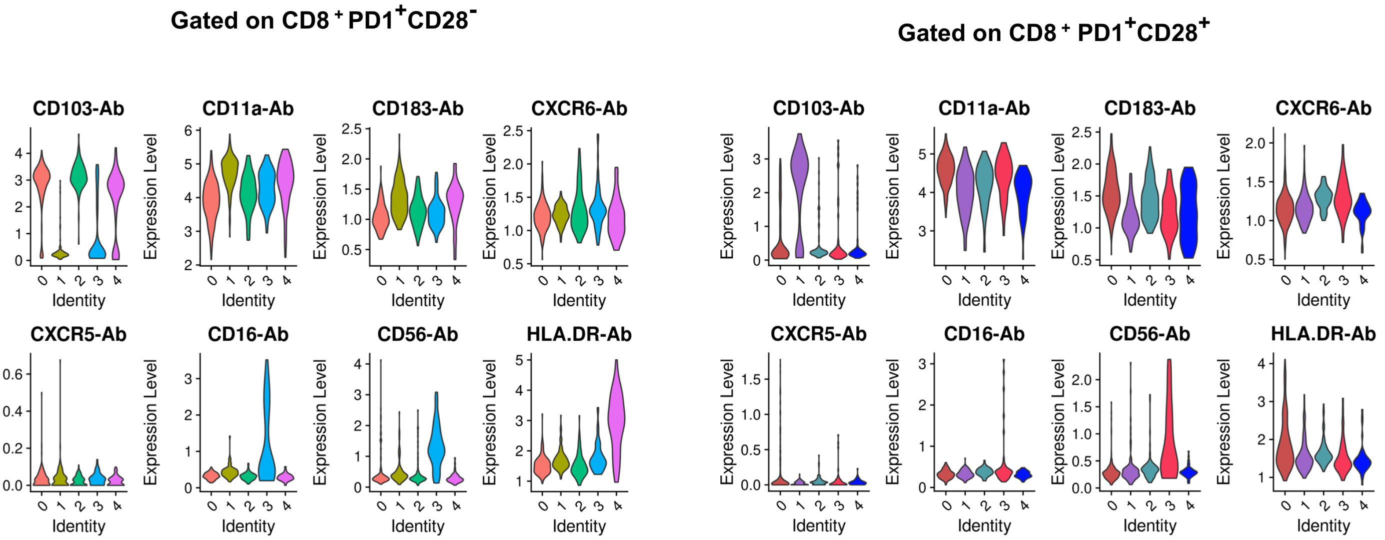

C

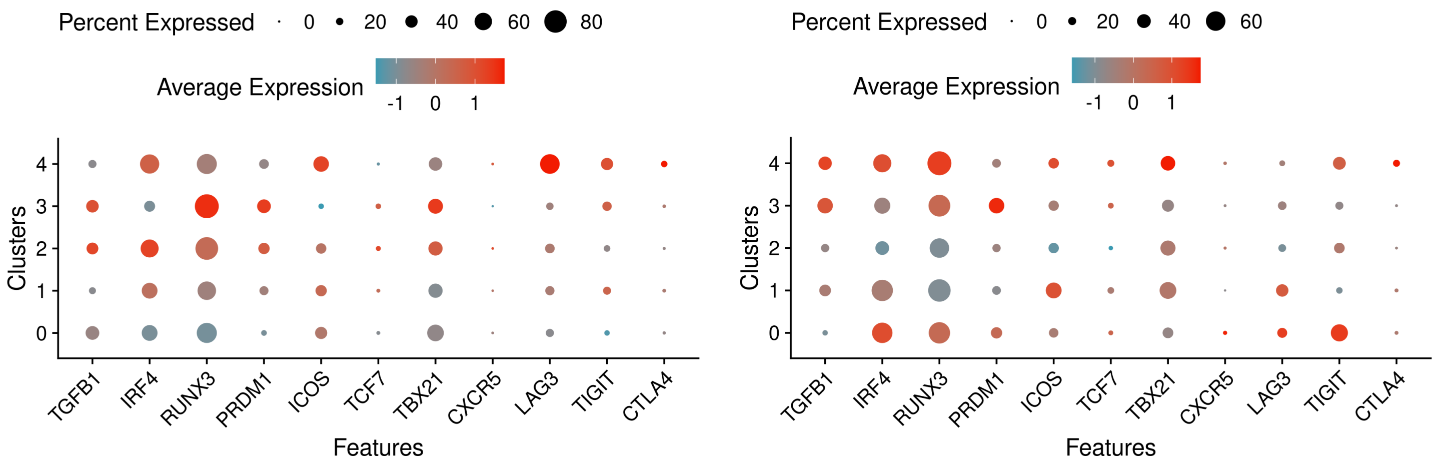

Supplement: Supplementary file 15 — Additional file 15: Figure S10. Expression of selected T-cell immune markers in the different CD8+ PD1+CD28− and PD1+CD28+ T-cell subsets. A UMAP showing the expression of selected mAbs from the implemented Immune Discovery Panel, in gated total live cells. B Violin-plot showing the expression level of selected mAbs in the clusters identified within PD1+CD28− and PD1+CD28+ T-cell subsets. C Heatmap-dot plot showing the percentage and the expression level of selected genes. [file 13046_2023_2846_MOESM15_ESM.pdf]
